# Tumor Immunophenotyping‐Derived Signature Identifies Prognosis and Neoadjuvant Immunotherapeutic Responsiveness in Gastric Cancer

**DOI:** 10.1002/advs.202207417

**Published:** 2023-03-30

**Authors:** Jia‐Bin Wang, Qing‐Zhu Qiu, Qiao‐Ling Zheng, Ya‐Jun Zhao, Yu Xu, Tao Zhang, Shuan‐Hu Wang, Quan Wang, Qin‐Wen Jin, Yin‐Hua Ye, Ping Li, Jian‐Wei Xie, Jian‐Xian Lin, Jun Lu, Qi‐Yue Chen, Long‐Long Cao, Ying‐Hong Yang, Chao‐Hui Zheng, Chang‐Ming Huang

**Affiliations:** ^1^ Department of Gastric Surgery Department of General Surgery Fujian Medical University Union Hospital Fuzhou 350001 P. R. China; ^2^ Ministry of Education Key Laboratory of Gastrointestinal Cancer (Fujian Medical University) Fujian Medical University Fuzhou 350122 P. R. China; ^3^ Department of Medical Microbiology Fujian Key Laboratory of Tumor Microbiology Fujian Medical University Fuzhou 350122 P. R. China; ^4^ Department of Pathology Fujian Medical University Union Hospital Fuzhou 350001 P. R. China; ^5^ Department of Gastrointestinal Surgery West District of the First Affiliated Hospital of University of Science and Technology of China Hefei 230031 P. R. China; ^6^ Department of Gastrointestinal and Hernia Surgery The First Affiliated Hospital of Kunming Medical University Kunming 650032 P. R. China; ^7^ Department of Gastrosurgery Liaoning Cancer Hospital & Institute Cancer Hospital of China Medical University Shenyang 110042 P. R. China; ^8^ Department of Gastrointestinal Surgery The First Affiliated Hospital of Bengbu Medical College Bengbu 233004 P. R. China; ^9^ Department of Gastrointestinal Surgery The First Hospital of Jilin University Changchun 130061 P. R. China; ^10^ Department of Gastrointestinal Surgery Guangxi Medical University Affiliated Tumor Hospital Nanning 530021 P. R. China

**Keywords:** gastric cancer, immune contexture, neoadjuvant immune checkpoint inhibitor therapy, prognosis, tumor microenvironment

## Abstract

The effectiveness of neoadjuvant immune checkpoint inhibitor (ICI) therapy is confirmed in clinical trials; however, the patients suitable for receiving this therapy remain unspecified. Previous studies have demonstrated that the tumor microenvironment (TME) dominates immunotherapy; therefore, an effective TME classification strategy is required. In this study, five crucial immunophenotype‐related molecules (WARS, UBE2L6, GZMB, BATF2, and LAG‐3) in the TME are determined in five public gastric cancer (GC) datasets (*n* = 1426) and an in‐house sequencing dataset (*n* = 79). Based on this, a GC immunophenotypic score (IPS) is constructed using the least absolute shrinkage and selection operator (LASSO) Cox, and randomSurvivalForest. IPS^Low^ is characterized as immune‐activated, and IPS^High^ is immune‐silenced. Data from seven centers (*n* = 1144) indicate that the IPS is a robust and independent biomarker for GC and superior to the AJCC stage. Furthermore, patients with an IPS^Low^ and a combined positive score of ≥5 are likely to benefit from neoadjuvant anti‐PD‐1 therapy. In summary, the IPS can be a useful quantitative tool for immunophenotyping to improve clinical outcomes and provide a practical reference for implementing neoadjuvant ICI therapy for patients with GC.

## Graphical abstract

In this study, an immunophenotypic score (IPS) is constructed to quantify the tumor microenvironment effectively in patients with gastric cancer (GC). In addition, the patients with GC and hypo‐IPS identified as immune‐activated status are evaluated and show a good prognosis and are suitable for neoadjuvant anti‐PD‐1 therapy.

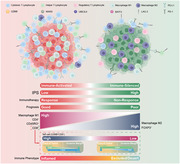

## 1 Introduction

Immune checkpoint inhibitors (ICIs) have emerged as a revolutionary approach to significantly improve cancer immunotherapy by targeting immune checkpoints.^[^ ^1^ ^]^ Many studies have reported the safety and efficacy of immune checkpoint therapy in treating gastrointestinal tumors.^[^ ^2^ ^]^ In the phase III KEYNOTE‐062 study, a monoclonal anti‐PD‐1 antibody showed single‐agent activity in patients with advanced gastric cancer (GC) with high microsatellite instability (MSI‐H).^[^ ^3^ ^]^ The anti‐PD‐1 antibody significantly improved progression‐free survival irrespective of PD‐L1 expression in the ATTRACTION‐4 trial.^[^ ^4^ ^]^ Additionally, an improvement in the overall survival (OS) of patients with a combined positive score (CPS) ≥5 combined with standard chemotherapy was observed in the global CheckMate‐649 trial and the ORIENT‐16 trial conducted in China.^[^ ^5^ ^]^ Thus, immunotherapy is the first line of treatment for GC,^[^ ^6^ ^]^ and a series of biomarkers represented by PD‐L1 have shown significant advantages in advanced or metastatic GC; however, the effect of treatment in resectable GC remains unknown.

Neoadjuvant immunotherapy is a potential treatment strategy for tumors that may be curable. More than 100 clinical trials of neoadjuvant anti‐PD‐(L)1 blockade (as monotherapy or combination therapy) are ongoing or planned for various tumor types.^[^ ^7^ ^]^ In the GERCOR NEONIPIGA phase II study, neoadjuvant anti‐PD‐1 therapy resulted in a complete pathological response in nearly 60% of patients with esophagogastric adenocarcinoma with MSI/mismatch repair deficient (dMMR),^[^ ^8^ ^]^ demonstrating that neoadjuvant therapy is effective and may cure. In another small phase II clinical trial, 12 patients with rectal cancer received novel PD‐1 blockade immunotherapy. Their tumors disappeared without follow‐up chemotherapy, radiation, or surgery.^[^ ^9^ ^]^ Neoadjuvant immunotherapy enhances systemic antitumor immunity and immune surveillance after surgery.^[^ ^10^ ^]^ Thus, screening patients suitable for neoadjuvant ICI therapy is necessary, which may be superior or complementary to the existing treatment options.

The benefits of ICI therapies depend primarily on the tumor microenvironment (TME) status, in which T cells are critical as antitumor executors. Some studies have classified tumors into “hot” (inflamed), “altered” (excluded/immunosuppressed), and “cold” (desert) phenotypes based on the spatial localization of T cells relative to the tumor and stromal compartments.^[^ ^11^ ^]^ “Hot” tumors may be more likely to respond to immune interventions to counteract the tumor‐induced T cell dysfunction. In contrast, “altered” and “cold” tumors may require novel targeted therapies to induce T cell activation and migration because of the lack of tumor‐infiltrating T cells in the core of the tumor (CT). Since antitumor immunity is a multi‐step complex process, the widespread intermolecular communication of other components within the TME cannot be ignored. Thorsson et al.^[^ ^12^ ^]^ identified six immune subtypes based on the communication and regulatory mechanisms of various immune components within the TME. This provides new insights into classifying multi‐characteristic immunophenotypes. Despite developing several TME signatures associated with response to ICI treatment, independent testing remained suboptimal, which may be attributed to strong inter‐ and intra‐tumor heterogeneity.^[^ ^13^ ^]^ Therefore, identifying more comprehensive and reliable TME classification strategies to determine the immunophenotype of TME immunotherapy response is essential to enhance antitumor immunity and improve clinical outcomes.

The immunophenotype suitable for immunotherapy, particularly neoadjuvant immunotherapy, remains unclear. Although MSI/TMB‐based staging is available in the immunotherapy of GC, robustness needs to be further enhanced. In this study, we aimed to identify the immunophenotypes associated with immunotherapy responsiveness in the TME and to construct a simple and reliable TME signature‐derived immunophenotyping for patients with GC to delineate the immune context, reveal prognostic information, and predict ICI treatment response. Unlike previous studies, the results of this study can be used to guide neoadjuvant immunotherapy and help surgeons select more favorable regimens for patients with GC based on immunophenotype.

## 2 Results

### 2.1 The Overall Design of this Study

**Figure 1:**
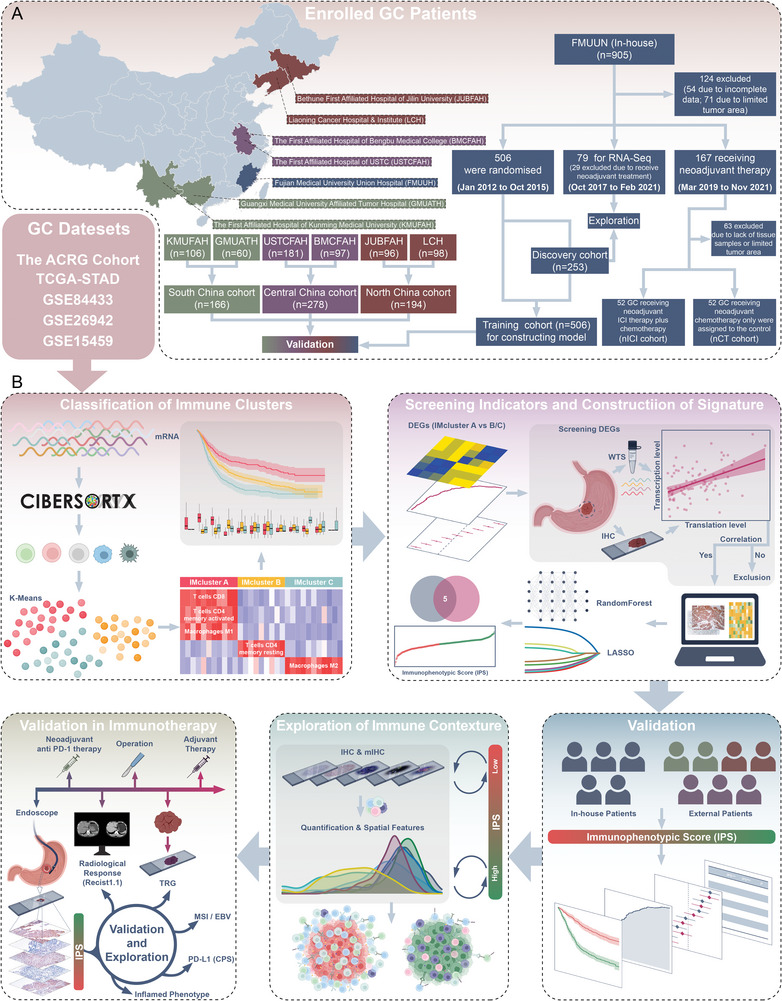
The overall design of the study. GC, Gastric Cancer; DEGs, Differentially Expressed Genes; WTS, Whole‐Transcriptome Sequencing; IHC, Immunohistochemistry; IPS, Immunophenotypic Score; mIHC, Multiplex Immunohistochemistry Staining; TRG, Tumor Regression Grade; CPS, Combined Positive Score.

**Figure** **1** illustrates the procedure used in this study.

### 2.2 Construction of an Immunophenotype‐Based Signature

In this exploratory study, we processed the gene expression profiles of 1426 patients with GC using bioinformatics algorithms to identify three clusters of prognosis‐ and immune checkpoint‐related immunophenotypes in TME (Figure S1A,B, Supporting Information). In brief, five immune cell types (memory‐activated CD4 T cells, M1 macrophages, CD8 T cells, M2 macrophages, and memory‐resting CD4 T cells) were screened for associations with immune checkpoint‐related genes and prognosis in the ACRG dataset (Figure S1A, Supporting Information). Based on the screened immune cell types, unsupervised cluster analysis was performed in each of the five GC datasets (ACRG/GSE66229, GSE84433, GSE26942, GSE15459, and TCGA‐STAD) to delineate immune clusters (Figure S1B, Supporting Information). IMcluster A exhibited immune activation, characterized by the hyperexpression of CD8 T cells, memory CD4 T cells, and M1 macrophages (Figure S1C,D, Supporting Information). Kaplan–Meier survival analysis showed that invasive margin (IM) cluster A was associated with an improved prognosis for patients with GC (Figure S1E, Supporting Information). The publicly available dataset supported the conclusion that IMcluster A represents a well‐prognoses and immune checkpoint‐related immunophenotype.

### 2.3 Construction of an Immunophenotype‐Based Signature Applicable to Pathological Tissues

The respective weights were assigned to the 497 differentially expressed genes (DEGs; IMcluster A vs IMclusterB/C) screened using the “limma” package. Based on the weight ranking, the top 30 DEGs were selected as the major immune activation genes (Figure S2A, Supporting Information). In addition to gene expression profile‐based signatures, pathological tissue‐applicable models have high detection convenience and predictive accuracy. However, IMcluster was determined at the transcriptional level. Immunohistochemical staining of pathological tissue detected the translational level. To determine whether these immune activation genes screened at the transcriptional level can be used for immunohistochemical detection, transcriptome sequencing was performed to obtain the mRNA expression profiles of the top 30 DEGs in 79 gastric adenocarcinoma tissues from our center. Additionally, the pathological tissues of these 79 patients with the top 30 DEGs were stained using immunohistochemistry. The correlation between each gene's transcriptional and translational levels was calculated using Spearman's test (Figure S2B, Supporting Information). Eight of the top 30 DEGs (GZMB, WARS, LAG‐3, ETV7, BATF2, PSMB9, PSMB10, and UBE2L6) were selected according to their *p*‐value.

Immunohistochemical staining of these eight genes was performed using the discovery cohort (Figure S3A, Supporting Information). The least absolute shrinkage and selection operator (LASSO) Cox and random survival forest algorithms were used to screen five molecules (GZMB, WARS, LAG‐3, BATF2, and UBE2L6) and were found to be strongly associated with the prognosis of GC. Subsequently, a proportional risk regression model based on five indicators, called the immunophenotypic score (IPS), was constructed. Patients with GC were classified into low‐(IPS^Low^) and high‐ (IPS^High^) risk groups according to their risk scores. The detailed steps of the process are described in the section, ‘Construction of the IPS’, Experimental Section.

### 2.4 Characterization of the Transcriptome and Genome of the IPS

Simultaneous evaluation of IMcluster and IPS was possible only in the patients of the FMUUN_RNA‐Seq cohort. We found that 78.1% of IMcluster A patients were identified as IPS^Low^, while IPS^High^ was mainly composed of IMcluster B and C by comparing the patient composition of IMcluster and IPS (Figure S3F, Supporting Information). For IMcluster, the clustering results were consistent with those obtained from the five public datasets (Figure S4A, Supporting Information). IPS^Low^ was found to have most of the features of IMcluster A, whereas IPS^High^ had the opposite. The transcriptomic and genomic features of the FMUUN_Seq cohort were analyzed because the biological properties of different IPSs are different. GO enrichment analysis of the DEGs between IPS^Low^ and IPS^High^ revealed that genes implicated in immune activation were enriched in IPS^Low^ (Figure S4B,C, Supporting Information). GSEA revealed that immune activation, immunotherapeutic response, antigen presentation, and tumor‐killing‐related pathways were upregulated in IPS^Low^ (Figure S4D, Supporting Information). Furthermore, whole‐exome sequencing of 48 cases in the FMUUN_Seq cohort was performed simultaneously, and it was found that IPS^Low^ was consistent with significantly more tumor mutations than IPS^High^ (Figure S4E, Supporting Information). By analyzing mutation annotation files, mutated genes were found to differ between IPS^Low^ and IPS^High^. This may provide new perspectives on the formation of immunophenotypic disparities (Figure S4F, Supporting Information). In summary, IPS^Low^ shares most of the overlap with IMcluster A. Furthermore, transcriptomic and genomic profiles indicated that IPS^Low^ featured a higher tumor mutational load associated with immune activation and an improved immunotherapeutic response.

### 2.5 Validation of the Prognostic Presentation and Clinical Features of the IPS

**Figure 2:**
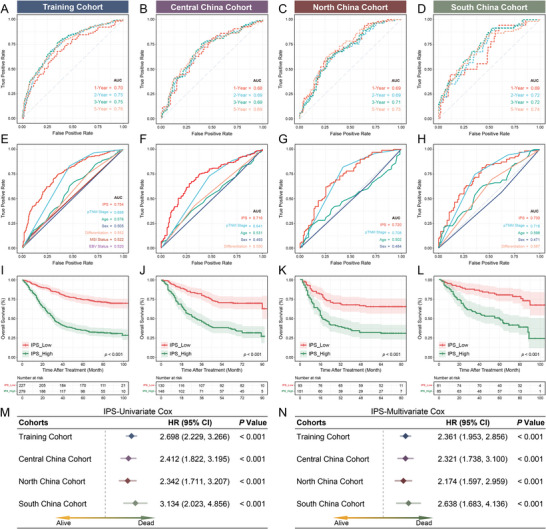
Data from four cohorts consisting of seven independent medical centers confirm the prognostic value of IPS for GC (A–D). Time‐dependent ROC curves of four cohorts demonstrate the accuracy and stability of IPS in predicting the prognosis. E–H) Comparison of the prognostic value of the IPS versus clinicopathological features in four cohorts by ROC curves. I–L) Kaplan–Meier curves for OS according to the IPS in four cohorts (log‐rank test, all *p* < 0.001). M,N) Univariate and multivariate Cox regression analysis was performed to explore the prognostic value of IPS (all *p* < 0.001). Variables with statistical significance in the univariate analysis were integrated into the multivariate analysis. In addition, the results of other clinicopathological variables are presented in Figure S6, Supporting Information. The dotted line represents the hazard ratio (HR) = 1. Training Cohort: *n* = 506, Central China Cohort: *n* = 178, North China Cohort: *n* = 194, South China Cohort: *n* = 166.

To further validate the performance of the IPS for clinical translation, the prognostic potential of the IPS was examined in the training cohort and three external cohorts consisting of patients with GC from six external independent medical centers. Time‐dependent ROC curves revealed the IPS's robust and stable discriminatory power in four independent cohorts (**Figure** **2**A–D). Previous studies have reported that some clinical features (e.g., AJCC_8th_ and differentiation) and molecular subtypes (MSI and Epstein–Barr virus [EBV] status) can be used to evaluate the prognosis of patients with GC. Therefore, the efficacy of IPS was compared with that of other clinical features or molecular subtypes in predicting the prognosis. As shown in Figure 2E–H, the predictive accuracy of the IPS was significantly superior to that of other variables including the AJCC_8th_, age, sex, differentiation, MSI status, and EBV status. In addition, Kaplan–Meier survival analysis revealed that patients with IPS^Low^ had better OS than those with IPS^High^ in the four independent cohorts (Figure 2I–L). Stratified analysis based on AJCC_8th_, differentiation, MSI status, and EBV status showed the same trend (Figure S5A–D, Supporting Information). The lack of statistical significance for stage I in the training cohort and the Central China cohort and stage II in the North China cohort may be attributed to the smaller sample size and lower mortality rates. Univariate and multivariate Cox regression analyses of multiple cohorts confirmed the prognostic value of the IPS. The IPS and AJCC_8th_ were significant in multivariate Cox analysis, indicating that the IPS combined with AJCC is an excellent combination for predicting the prognosis (Figure 2M,N and Figure S6, Supporting Information). Furthermore, *χ* ^2^ tests were used to determine the association between the IPS, clinicopathological features, and molecular subtypes. The results showed a significant correlation between IPS and AJCC_8th_, MSI status, and EBV status (Tables S1 and S2, Supporting Information). This indicated that IPS^Low^ had less lymph node metastasis and enhanced muted tumor invasion (Figure S7A–C, Supporting Information).

Overall, multicenter data supported IPS as a robust prognostic biomarker for GC, independent of clinicopathological features. Moreover, MSI‐H and EBV‐positive patients had a lower IPS, which could mean that the IPS is a potential surrogate for MSI or EBV status and that patients with hypo‐IPS may exhibit partial MSI‐H or EBV‐positive features (Figure S7D,E, Supporting Information). In previous studies, MSI‐H and EBV positivity have been considered favorable features for immune infiltration and immunotherapeutic responses,^[^ ^9^, ^14^ ^]^ reflecting the IPS's potential in immunotherapy. The enrichment analysis indicated that the IPS is related to the activation of antitumor immunity and the immunotherapeutic response (Figure S4C,D, Supporting Information). Therefore, it was hypothesized that the IPS could map the MSI/EBV status and more accurately reflect the immune status and response to immunotherapy.

### 2.6 Deconstructing the Immune Microenvironmental Landscape of IPS Specificity

**Figure 3:**
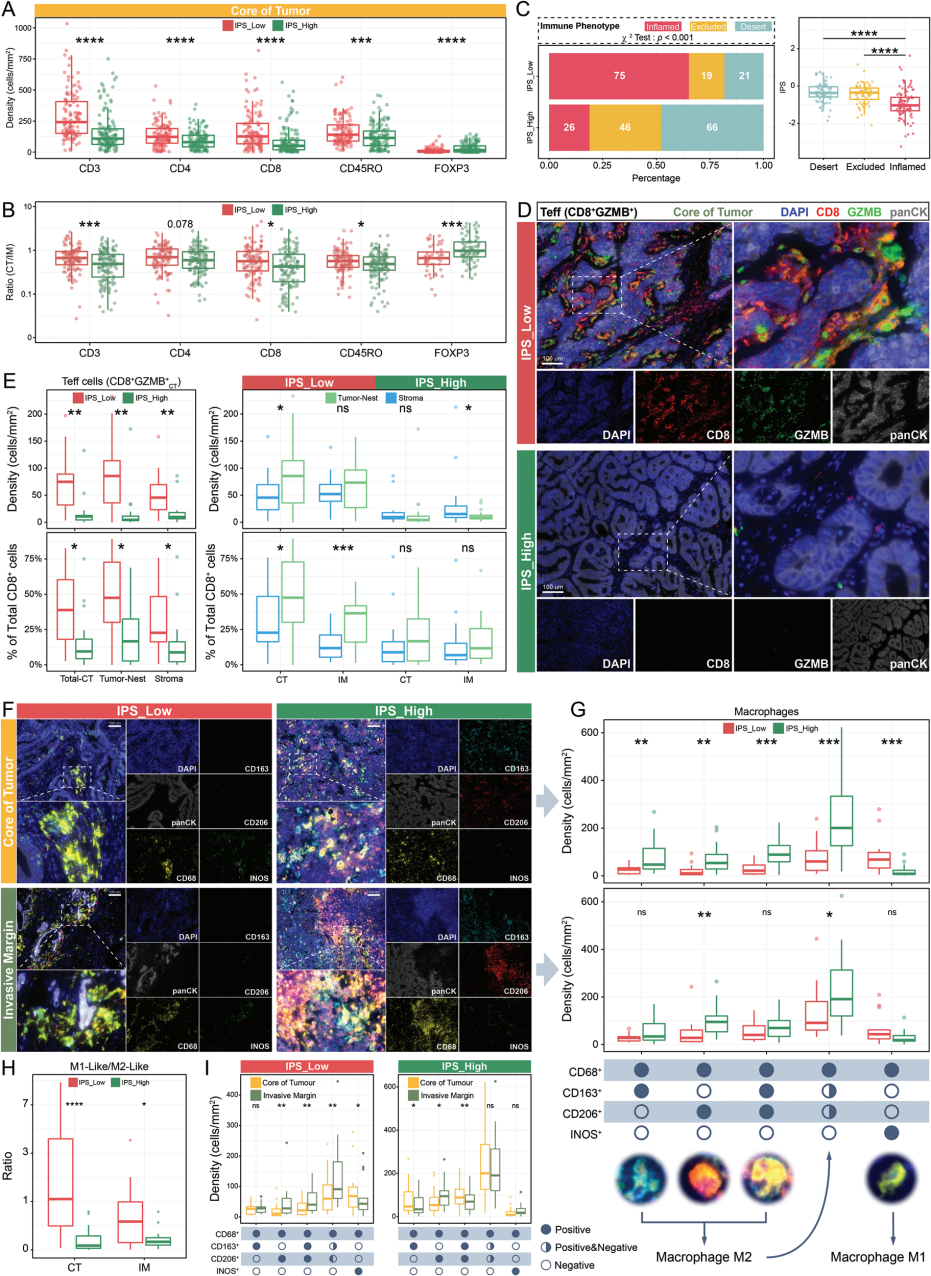
The IPS‐specific landscape of the tumor immune microenvironment. A) Comparison of immune infiltration in the core of the tumor (CT; CD3^+^, CD4^+^, CD8^+^, CD45RO^+^, and FOXP3^+^) between the IPS^Low^ and IPS^High^ in the Discovery Cohort (*n* = 253, IPS^Low^ = 115, IPS^High^ = 138). ***p < 0.001; ****p < 0.0001, Mann–Whitney U‐test. B) Comparing the ratio of immune cell infiltration (CD3^+^, CD4^+^, CD8^+^, CD45RO^+^, and FOXP3^+^) in the CT to the invasive margin (IM) between the IPS^Low^ and IPS^High^. Cases with density <5 cells/mm^2^ were excluded to reduce the abnormal oversize/undersize ratio (CD3^+^: *n* = 251, CD4^+^: *n* = 244, CD8^+^: *n* = 239, CD45RO^+^: *n* = 249, FOXP3^+^: *n* = 130). *p < 0.05; ***p < 0.001, Mann–Whitney U‐test. C) Components of the immunophenotypes (Inflamed, Excluded, and Desert) of IPS^Low^ versus IPS^High^ (*p* < 0.001, *χ* ^2^ test), while comparing the IPS between the three immunophenotypes. ****p < 0.0001, Mann–Whitney U‐test. D) Multiplexed immunohistochemical staining was used to visualize the effector T cells (Teffs; GZMB^+^CD8^+^) in the CT of IPS^Low^ versus IPS^High^, and panCK^+^ was used to segment the tumor nest and stroma (CD8‐red, GZMB‐green, panCK‐grey, and DAPI‐blue; *n* = 31; scale bar = 100 µm). E) Comparison of the density and ratio (to total CD8^+^ cells) of Teffs in the CT between IPS^Low^ and IPS^High^ (Mann–Whitney U‐test), and the distribution characteristics of Teffs in different locations of the tumor nest and stroma (Wilcoxon matched‐pairs signed rank test). IPS^Low^: *n* = 15, IPS^High^: *n* = 16; *p < 0.05; **p < 0.01; ***p < 0.001. F) Multiplex immunofluorescence staining characterized the macrophage infiltration profile of IPS^Low^ and IPS^High^ in the CT and IM (CD68‐yellow, CD163‐cyan, CD206‐red, INOS‐green, panCK‐grey, and DAPI‐blue; *n* = 31, scale bar = 100 µm). G) Comparison of the density of different macrophage subtypes between IPS^Low^ and IPS^High^ in the CT (upper panel) and IM (lower panel). The red dotted line represents the margin between the tumor and normal tissue (IPS^Low^: *n* = 15, IPS^High^: *n* = 16). *p < 0.05; **p< 0.01; ***p < 0.001; ****p < 0.0001, Mann–Whitney U‐test. H) Differences in the ratio of M1 to M2 macrophages in IPS^Low^ versus IPS^High^. Greater than 1 means more M1‐like, and less than 1 means more M2‐like (IPS^Low^: *n* = 15, IPS^High^: *n* = 16). *p < 0.05; ****p < 0.0001, Mann–Whitney U‐test. I) Distribution tendency of different macrophage subsets in IPS^Low^ and IPS^High^ tumors. *p < 0.05; ** *p* < 0.01, Wilcoxon matched‐pairs signed rank test. In all box plots of this figure, the thick line shows the median value. The bottom and top of the boxes are the 25th and 75th percentile (interquartile range) and extend through the whiskers to 1.5 times the interquartile range.

Several immune markers (including total CD45^+^ leukocytes, CD3^+^, cytotoxic CD8^+^, helper CD4^+^, activated and memory CD45RO^+^, and FOXP3^+^ regulatory T cells) were first quantified in the CT and IM to understand the IPS‐specific immune microenvironment (Figure S8A, Supporting Information). No difference in total leukocyte (CD45^+^) infiltration was observed between IPS^Low^ and IPS^High^ tumors (Figure S8B, Supporting Information). **Figure** **3**A and Figure S8C, Supporting Information, indicated that in the CT or IM, IPS^Low^ tumors exhibited more enriched infiltration of CD3^+^, CD4^+^, CD8^+^, and CD45RO^+^ compared to that in IPS^High^. In contrast, FOXP3^+^ cells showed more significant infiltration in IPS^High^ tumors. Spearman's correlation analysis revealed the same trends (Figure S8D, Supporting Information). By comparing the infiltration of CT and IM, it was discovered that CD3^+^, CD8^+^, and CD45RO^+^ had a higher core‐to‐margin ratio (CT/IM) in IPS^Low^ tumors, which seemed to imply a more intensive infiltration of CD3^+^, CD8^+^, and CD45RO^+^ from the IM toward the CT in IPS^Low^ tumors (Figure 3B). FOXP3^+^ was found to be silent on CT of IPS^Low^ tumors. The infiltration distribution characteristics of the respective IPS fit the description of the three previously reported immunophenotypes (inflamed, excluded, and desert). Therefore, the immunophenotypic compositions of different IPSs were evaluated and compared (Figure S8E, Supporting Information). As expected, 65.23% of tumors in IPS^Low^ were inflamed tumors, which was significantly higher than the 18.84% in IPS^High^ (*χ* ^2^ test; *p* < 0.001, Figure 3C). In contrast, excluded (33.33%) and desert (47.82%) tumors were more frequently found in the IPS^High^. The Kruskal–Wallis test confirmed that inflamed had the lowest IPS (Figure 3C). This may imply that the IPS reflects the spatial distribution characteristics of T cells to some extent. In addition, Teffs are critical for antitumor immunity as tumor‐killing executors, and their marker, GZMB, is one of the components of the IPS. Therefore, the association between Teffs and IPS was investigated by characterizing Teffs in the tumor nest versus the stroma using CD8A and GZMB (Figure 3D and Figure S8F, Supporting Information). The findings indicated that IPS^Low^ tumors had abundant infiltration of Teffs and concentrated Teffs in the tumor nest. This trend was more pronounced in the CT (Figure 3E and Figure S8G,H, Supporting Information). Moreover, a higher proportion of Teffs was observed in IPS^Low^ tumors and reached a maximum in the tumor nest on CT. In contrast, Teffs in IPS^High^ were localized in the stroma. These data suggest that IPS^Low^ is a subtype with a positive antitumor immune response.

The clustering basis of the IMcluster on which the IPS was developed contained M1‐ and M2‐like macrophages. Therefore, the infiltration characteristics of TAMs from diverse IPS tumors were explored (Figure 3F). Multiplex immunofluorescence staining demonstrated distinct infiltration profiles of TAMs in IPS^Low^ versus IPS^High^ tumors. IPS^High^ tumors show a significant abundance of M2‐like TAMs in the CT and IM of the tumor compared with IPS^Low^ tumors (Figure 3G,H). In contrast, in IPS^Low^ tumors, a more M1‐like phenotype was observed on CT (Figure 3G,H). Although a trend was observed for CD68^+^CD163^+^, CD68^+^CD163^+^CD206^+^, and CD68^+^INOS^+^ in the IM, the results were not significant, which may be related to the limited sample size. Moreover, the distribution of each TAM phenotype in IPS^Low^ and IPS^High^ tumors was explored. In IPS^Low^ tumors, CT revealed an increased density of CD68^+^INOS^+^ M1‐like TAMs compared to the IM, while CD68^+^CD206^+^ and CD68^+^CD163^+^CD206^+^ M2‐like TAMs tended to accumulate in the IM, with no discrepancy for CD68^+^CD163^+^ (Figure 3I). For IPS^High^ tumors, aggregation in the CT on TAMs of CD68^+^CD163^+^ was observed, whereas the distribution of TAMs of CD206 was reversed (Figure 3I).

In conclusion, M1‐like TAM levels were more intense in the CT of IPS^Low^ tumors. They decreased toward the IM, whereas M2‐like (CD68^+^CD163^+^ and CD68^+^CD163^+^CD206^+^) TAM levels were less dense and restricted to the tumor margins. In IPS^High^ tumors, TAMs had a more M2‐like phenotype than in IPS^Low^ tumors, with CD68^+^CD163^+^ and CD68^+^CD163^+^CD206^+^ M2‐like TAMs accumulated in the CT and decreased toward the IM, and CD68^+^CD206^+^ M2‐like TAMs accumulated in the IM and decreased toward the CT.

Evaluation of the immune microenvironment indicated that the antitumor effect of the TME of IPS^High^ was muted with a significant inhibitory profile, which was defined as immune‐silenced. Conversely, IPS^Low^ exhibited an immune‐activated profile, which was considered a beneficial signal for immunotherapy.^[^ ^15^ ^]^ Therefore, we hypothesized that different levels of IPSs would respond differently to immunotherapy.

### 2.7 IPS Predicts Neoadjuvant Immunotherapeutic Benefits

**Figure 4:**
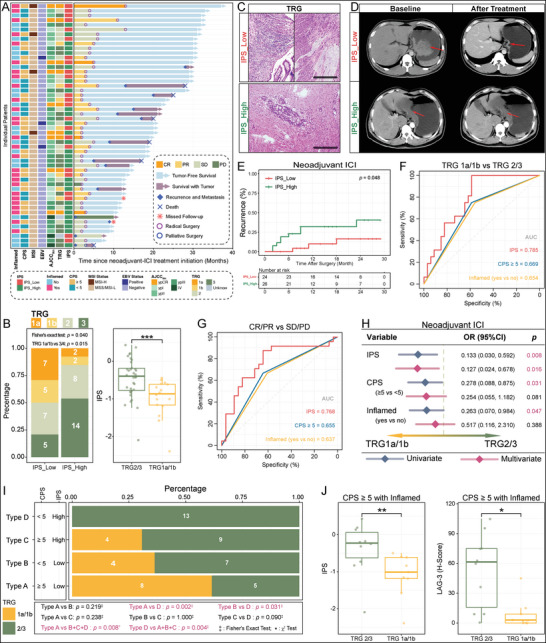
IPS accurately predicts the neoadjuvant ICI therapy response. A) Overview of treatment for patients with locally advanced GC receiving neoadjuvant ICI therapy (*n* = 52). B) Composition of TRG to neoadjuvant ICI therapy in IPS^Low^ (*n* = 24) versus IPS^High^ (*n* = 26; *p* = 0.040, Fisher's exact test). Moreover, the IPS was compared between TRG 1a/1b and TRG 3/4 patients (*p* < 0.001, Mann–Whitney U‐test). The thick line shows the median value. The bottom and top of the boxes are the 25th and 75th percentile (interquartile range) and extend through the whiskers to 1.5 times the interquartile range. C) Postoperative pathological tissue images of no. 40 (upper panel, IPS^Low^) and no. 47 (lower panel, IPS^High^). Patient no. 40 had a completely regressed tumor (TRG1a), while patient no. 47 still had a residual tumor (TRG 3). Scale bar = 50 µm. D) CT imaging changed before and after neoadjuvant ICI therapy in patient no. 40 (IPS^Low^) and patient no. 47 (IPS^High^). E) Kaplan–Meier survival analysis demonstrated recurrence in IPS^Low^ versus IPS^High^ patients (*p* = 0.048, log‐rank test). F,G) Comparing the accuracy of biomarkers (IPS, CPS, and Inflamed phenotype) in predicting the response to neoadjuvant ICI therapy by ROC curves. H) Univariate and multivariate logistic regression analysis to confirm the value of biomarkers (IPS, CPS, and Inflamed phenotype) for predicting neoadjuvant ICI therapy (outcome: TRG1a/1b). OR: Odd Ratio. I) Comparison of the TRG to neoadjuvant ICI therapy across Type A (IPS^Low^ with CPS ≥ 5), Type B (IPS^Low^ with CPS < 5), Type C (IPS^High^ with CPS ≥ 5), and Type D (IPS^High^ with CPS < 5). J) Comparison of IPS and LAG‐3 in TRG1a/1b (*n* = 9) and TRG2/3 (*n* = 10) patients with GC with CPS ≥ 5 and inflamed phenotype (*n* ^total^ = 19). ***p < 0.001, Mann–Whitney U‐test. The thick line shows the median value. The bottom and top of the boxes are the 25th and 75th percentile (interquartile range) and extend through the whiskers to 1.5 times the interquartile range.

In patients receiving neoadjuvant therapy prior to treatment, we had only biopsied tissue to evaluate. In this study, we demonstrated that IPS could be used for biopsy tissue that can be evaluated for tumor areas larger than 0.16 mm^2^ (Figure S9, Supporting Information, and ‘Identification of IPS Applicability on Gastroscopic Biopsy Specimens’, Experimental Section). When the IPS of 52 patients who received anti‐PD‐1 therapy was evaluated, 25 were classified as IPS^Low^ and 27 as IPS^High^ (**Figure** **4**A and Figure S10A, Supporting Information). IPS^Low^ patients experienced more tumor regression after neoadjuvant immunotherapy than IPS^High^ patients (TRG1a/1b: IPS^Low^ = 50%, IPS^High^ = 15.4%; Fisher's exact test: *p* = 0.015; Figure 4B,C). In addition, the Mann–Whitney test confirmed a lower IPS in patients with TRG 1a/1b (*p* = 0.0009; Figure 4B). We analyzed the radiological results. Among patients on IPS^Low^, three patients had complete tumor disappearance (CR) and 14 patients had partial remission, resulting in an objective response rate (ORR) of 68%, which was significantly better than the ORR of 25.9% among IPS^High^ patients (Fisher's exact test: *p* = 0.009; Figure 4D and Figure S10B, Supporting Information). In parallel, Kaplan–Meier survival analysis revealed that patients with IPS^Low^ tumors had a lower postoperative recurrence rate and longer recurrence‐free survival (RFS) than patients with IPS^High^ tumors (log‐rank *p* = 0.044; Figure 4E). In contrast, patients with IPS^High^ showed no changes before and after treatment. The *χ* ^2^ and Fisher's exact tests indicated that patients with IPS^Low^ had lower ypT, ypN, and ypTNM stages after receiving neoadjuvant immunotherapy (**Table** **1**).

**Table 1:** Comparing the efficacy of neoadjuvant ICI therapy combined with chemotherapy in patients with GC with IPS^Low^ versus IPS^High^

Characteristic	IPS^Low^		IPS^High^		*p*
	*n*	[%]	*n*	[%]	
Total Patients	25		27		
Response					0.002^a^ ^)^
CR/PR	17	68%	7	25.93%	
SD/PD	8	32%	20	74.07%	
TRG					0.015^b^ ^)^
1a/1b	12	48%	4	14.81%	
2/3	12	48%	22	81.48%	
ypT Stage					0.032^b^ ^)^
T0/T1	11	44%	4	14.81%	
T2/T3	13	52%	21	77.78%	
ypN Stage					0.003^a^ ^)^
N0	18	72%	8	29.63%	
N1‐N3	6	24%	17	62.96%	
ypTNM Stage					0.007^b^ ^)^
pCR/I	13	52%	4	14.81%	
II/III	12	48%	23	85.19%	

^a)^ Calculated by the Chi‐Square test

^b)^ Calculated by Fisher's exact test.

However, patients receiving neoadjuvant ICI therapy also received chemotherapy, which may have biased the results; thus, we included 52 patients who received only the neoadjuvant chemotherapy regimen as controls (Figure S10C and Table S3, Supporting Information). Among all IPS^Low^ patients who received ICI therapy versus those who received only chemotherapy, ICI therapy demonstrated a higher ORR, more significant tumor regression, and lower postoperative staging (ORR: nICI with nCT = 68%; nCT only = 42.3%; Table S4, Supporting Information). Although lacking statistical significance due to the limited sample size, this trend indicated that the predictive power of the IPS might be specific to neoadjuvant immunotherapy and independent of neoadjuvant chemotherapy. This further suggests that the IPS may remain applicable when anti‐PD‐1 therapy is co‐applied with other therapies such as different regimens of chemotherapy or molecular‐targeted therapies.

Patients with MSI‐H or EBV are considered suitable for immunotherapy; however, some non‐MSI‐H and non‐EBV patients could benefit from immunotherapy.^[^ ^16^ ^]^ The results of this study show that the IPS identifies beneficiaries of neoadjuvant immunotherapy in MSS (ORR: IPS^Low^ = 65.2%, IPS^High^ = 24%; *χ* ^2^ test *p* = 0.004) and EBV‐negative (ORR: IPS^Low^ = 65.2%, IPS^High^ = 26.9%; *χ* ^2^ test *p* = 0.007) patients (Figure S10D,E, Supporting Information); thus, potential immunotherapy strategies for MSS and EBV‐negative patients may be sought. In summary, the IPS may be an alternative to MSI or EBV subtypes and a powerful complement to MSI and EBV subtypes in immunotherapy applications. However, the limited sample of the nICI cohort, with only four patients with GC detected as MSI‐H (7.7%) and three as EBV‐positive (5.8%), limited further exploration.

Tumors with CPS ≥ 5 and the inflamed phenotype are vital signs of a positive response to anti‐PD‐L1 treatment;^[^ ^17^ ^]^ thus, the accuracy of the IPS with CPS and the inflamed phenotype in predicting anti‐PD‐1 therapy was compared by plotting ROC curves. The results showed that the AUC of the IPS was significantly better than that of the CPS and the inflamed phenotype, regardless of whether the radiological response or TRG was used as the outcome (Figure 4F,G). Univariate and multivariate logistic regression analyses supported the strong correlation between the IPS and the immunotherapeutic response (Figure 4H and Figure S10F, Supporting Information). To confirm whether the combination of IPS and CPS enhances the discrimination of the response to ICI therapy, patients were classified into four types according to the IPS and CPS. Patients with Type A (IPS^Low^ with CPS ≥ 5) had an ORR of 84.6% and a TRG1a/1b ratio of 61.5%. They were the group most likely to benefit from ICI therapy, whereas Type D (IPSHigh with CPS < 5) had an ORR of only 14.3% and a TRG1a/1b ratio of 61.5%. These patients were considered unsuitable for ICI therapy (Figure 4I and Figure S10G, Supporting Information). Survival analysis demonstrated that CPS combined with IPS significantly affected the long‐term prognosis of patients receiving neoadjuvant ICI therapy (Figure S10H, Supporting Information). In addition, ineffective ICI therapy was observed in 47.4% of patients with CPS ≥ 5 and an inflamed phenotype. Patients who failed to respond to ICI therapy had a higher IPS and a significant upregulation of LAG‐3 (Figure 4J and Figure S10I, Supporting Information). According to the results of this study, patients with the inflamed phenotype did not respond to anti‐PD‐1 therapy as consistently as expected, and non‐responders were still observed in patients with both the inflamed phenotype and PD‐L1 dual positivity. The IPS could identify these non‐responders. Herein, the association of IPS with the inflamed phenotype was identified, and it currently appears that IPS^Low^ potentially serves as an alternative to the inflamed phenotype in neoadjuvant ICI therapy.

Overall, IPS was a robust biomarker for predicting the response to ICI therapy by PD‐L1 and the inflamed phenotype. The combination of IPS and CPS accurately identified immunotherapy‐sensitive and ‐naïve patients. Additionally, overexpression of LAG‐3 in non‐responders with CPS > 5 and the inflamed phenotype was observed, raising concerns.

## 3 Discussion

**Figure 5:**
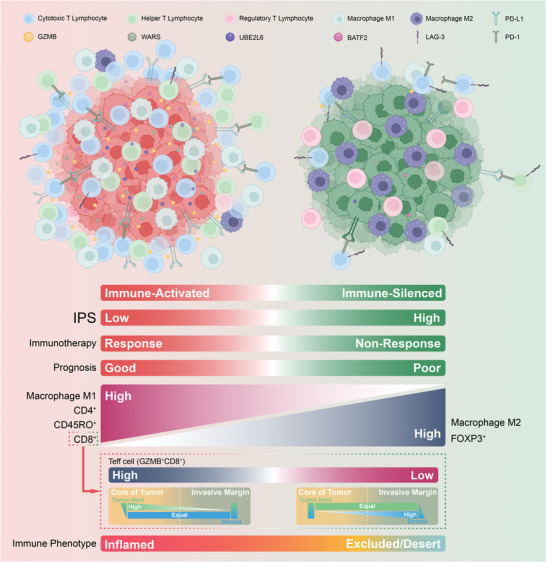
Schematic illustration of the characteristics associated with the immunophenotypic score (IPS) in this study.

Based on screened immunophenotypic characteristics, this study developed an IPS applicable to pathological tissues for more efficient quantification and classification of the TME in the clinical process. Patients with GC were classified into two immune states based on their IPS scores: IPS^Low^ matched immune‐activated, and IPS^High^ matched immune‐silenced. The two immune states of patients with GC were distinctly different regarding prognosis and responsiveness to ICI therapy, as supported by data from seven independent medical centers. **Figure** **5** illustrates the characteristics of patients with different IPS values in this study.

This promising performance of the IPS is attributed to its ability to separate the TME. In IPS^Low^ tumors, CD8^+^ and CD4^+^ T cells are abundantly recruited in the tumor parenchyma. Further analysis revealed that dense GZMB^+^CD8^+^ Teffs were present in the tumor nest region of the CT in IPS^Low^, reflecting the advantage of the tumor‐targeted migration ability of Teffs.^[^ ^18^ ^]^ In addition, M1 macrophages (CD68^+^INOS^+^) were found in high numbers in the tumor epithelium, together with Teffs defined, the hyperinflammatory microenvironment of IPS^Low^ tumors and a favorable outcome. These results suggest pre‐existing antitumor immunity, a prerequisite for immunotherapy efficacy.^[^ ^15^, ^19^ ^]^ Although previous studies have reported an increase in immune checkpoints and Tregs in the inflammation‐promoting environment through negative immune feedback,^[^ ^20^ ^]^ the opposite trend of Tregs was observed in this study, where Tregs appeared to be suppressed in the tumor core of IPS^Low^, which seems to indicate a hypoimmunosuppressive profile of IPS^Low^. In terms of immune checkpoints, coinciding with previous studies, IPS^Low^ tumors showed enriched expression of immune checkpoints, a self‐balancing of the immune system that is not driven by tumor cells.^[^ ^20b^ ^]^ Therefore, immunotherapy is expected to accelerate the immune effect and abrogate the tumor by disrupting this balance in the tumor parenchyma.

Neoadjuvant immunotherapy applied to resectable tumors has been shown to improve RFS and OS in several clinical trials. However, the known PD‐L1, MSI, EBV status, and TMB remain unstable in predicting the benefit of ICI therapy.^[^ ^16^, ^21^ ^]^ To date, effective biomarkers to predict the responsiveness to neoadjuvant ICI therapy before treatment have remained absent, particularly in GC. In this study, we revealed the predictive value of the IPS for neoadjuvant immunotherapy in GC, which could identify beneficiaries of non‐MSI‐H and EBV‐negative subtypes. In parallel, the predictive abilities of the IPS for neoadjuvant ICI therapy were superior to and independent of the two accepted biomarkers, PD‐L1 (CPS) and CD8 (inflamed phenotype). Moreover, the results of multivariate logistic regression indicated the potential of IPS in combination with PD‐L1. Patients with IPS^Low^ and CPS ≥ 5 benefitted from neoadjuvant ICI therapy. In contrast, patients with IPS^High^ and CPS < 5 were non‐responders to neoadjuvant ICI therapy and prone to postoperative relapse. Considering potential immune‐related adverse events, patients with IPS^High^ and CPS < 5 may be unsuitable for neoadjuvant ICI therapy.

PD‐L1‐rich and inflamed tumors are considered sensitive to ICI therapy;^[^ ^17^ ^]^ however, non‐responders remain observable in these tumors. Most of these non‐responders exhibited significantly elevated IPS. Further investigation revealed that the elevated IPS in these non‐responders was driven by LAG‐3 enrichment. Recent studies suggest that a single PD‐1/CTLA‐4 blockade therapy may not rescue the antitumor effects of T cells with high expression of LAG‐3.^[^ ^22^ ^]^ Based on this, we hypothesized that receiving a combination of LAG‐3 and PD‐1 blockade may be the key to reactivating the antitumor effect in these patients (IPS^High^, CPS ≥ 5, and inflamed phenotype). Results from several clinical studies have revealed the clinical benefit and safety of anti‐LAG‐3 in combination with anti‐PD‐1 for the treatment of a variety of solid tumors,^[^ ^23^ ^]^ which drove the FDA to approve Opdualag, the first combination of LAG‐3 and PD‐1 blockade.^[^ ^24^ ^]^ Moreover, a combination of relatlimab (anti‐LAG‐3) enhanced the effect of neoadjuvant anti‐PD‐1 therapy.^[^ ^25^ ^]^ While these results are encouraging, the effectiveness of the LAG‐3/PD‐1 combination inhibitor remains limited in unselected patients,^[^ ^26^ ^]^ and robust biomarkers are required to identify patients who could benefit. Compared with other TME‐based signatures, the IPS incorporated the contribution of LAG‐3, which may be a potential advantage of the IPS in predicting the combination of LAG‐3 and PD‐1 blockade therapy. We currently have no clinical evidence to confirm the value of the IPS in combination therapies, and a prospective multicenter trial is needed to validate our conjecture.

The IPS differs from other TME‐based signatures in that IPS is applicable not only to postoperative pathology specimens but also to preoperative biopsy specimens obtained preoperatively by gastroscopy, which facilitates the implementation of clinical translation. When patients are diagnosed with GC by biopsy tissue obtained by gastroscopy, only five indicators (WARS, UBE2L6, GZMB, BATF2, and LAG‐3) of immunohistochemical staining of the remaining biopsy tissue are sufficient to obtain the IPS to determine the strategy of neoadjuvant therapy without additional invasive examinations. Although the IPS holds significant clinical promise for GC, this study has some limitations. First, this study was based on retrospective data, and there might have been an unintentional bias in selecting patients with GC. Second, the limited sample size (FMUUN‐RNA_Seq Cohort) may have excluded some meaningful genes when selecting DEGs by confirming transcription‐translation concordance. Finally, the number of patients in the neoadjuvant ICI therapy cohort remained low, and a higher number of individuals in both the trial and control cohorts is necessary to confirm the results of this study.

## 4 Conclusion

In summary, the IPS is a robust and stable signature applicable to pathological tissues to evaluate the prognosis and response to neoadjuvant ICI therapy in patients with GC by comprehensively classifying the TME (Figure 5). Furthermore, the stratification of patients with GC by the IPS and CPS may be a valuable step toward more tailored and precise immunotherapy.

## 5 Experimental Section

### Study Design and Data Sources

In this multicenter, retrospective study, genomic data and clinicopathological information of 1426 patients with GC were obtained from five publicly available GC datasets, and 1326 patients with GC were enrolled from seven independent medical centers in different geographic regions of China (Figure 1A).

To identify potential genes that classify the TME, all gene expression data and clinicopathological information from TCGA‐STAD, ACRG/GSE66229, GSE84433, GSE26942, and GSE15459 were obtained from The Cancer Genome Atlas (TCGA) and Gene Expression Omnibus (GEO). For microarray data from GEO, the robust multiarray averaging (RMA) algorithm in the “Affy” package of the R software (version 3.6.3) was used to process raw data from the Affymetrix platform, and the “lumi” package was used to process raw data from the Illumina platform. For the TCGA‐STAD dataset, RNA‐Seq data (FPKM values) were transformed into transcripts with per‐kilobase million (TPM) values. Data from each of the five publicly available datasets were analyzed separately.

To construct and explore the TME‐based IPS, formalin‐fixed paraffin‐embedded (FFPE) specimens from 687 patients with GC from the Fujian Medical University Union Hospital (FMUUH, Fuzhou, China) were included. A total of 506 patients without neoadjuvant treatment who underwent D2 GC radical surgery between 2012 and 2015 were enrolled to construct and validate the prognostic value of the IPS, of which 253 were used to characterize the immune microenvironment. A total of 638 GC tissues and clinicopathological specimens collected between September 2008 and March 2016 from six external centers were used to test the prognostic value of the IPS. Of these, 98 patients were from the Liaoning Cancer Hospital & Institute (LCH, Shenyang, China), 96 from the Bethune First Affiliated Hospital of Jilin University (JUBFAH, Changchun, China), 97 from the First Affiliated Hospital of Bengbu Medical College (BMCFAH, Bengbu, China), 181 from the First Affiliated Hospital of the University of Science and Technology of China (USTCFAH, Hefei, China), 60 from the Guangxi Medical University Affiliated Tumor Hospital (GMUATH, Nanning, China), and 106 from the First Affiliated Hospital of Kunming Medical University (KMUFAH, Kunming, China). As illustrated in Figure 1A, the six independent medical centers were combined into three cohorts based on their geographical location. Clinicopathological information about the patients with GC enrolled in these cohorts is listed in Tables S5 and S6, Supporting Information. The inclusion criteria were as follows: 1) histological identification of GC, 2) no other malignant tumors or distant metastases, 3) availability of follow‐up data and clinicopathological characteristics, and 4) TNM staging of GC tumors according to the 2010 International Union Against Cancer guidelines. The exclusion criteria were: 1) death within 1 month of surgery, and 2) chemotherapy or radiotherapy before surgery. All participants with advanced GC routinely received fluorine‐based chemotherapy. The patient follow‐up strategy was available in our previous study (2). Informed consent was obtained from all the participants.

To explore the genomic features of IPS, 79 patients who underwent D2 GC radical surgery at FMUUH from 2017 to 2021 were included. Of these, 47 were commissioned by Novogene Co., Ltd. (Beijing, China) for whole‐exome and whole‐transcriptome sequencing, and 32 were sequenced by Kangchen Biotechnology Co., Ltd. (Shanghai, China) for whole‐transcriptome sequencing. Whole‐exome sequencing was performed on an Illumina HiSeq PE150 using an Agilent SureSelect Human All Exon V5/V6 (Agilent Technologies, CA, USA). Whole‐transcriptome sequencing was performed on an Illumina HiSeq platform using the TruSeq SR Cluster Kit v3‐cBot‐Hs (Illumina, CA, USA). After reducing batch effects by the “ComBat” algorithm, the FMUUH_RNA‐Seq cohort was assembled from the sequencing data analyzed by the two companies. The clinical characteristics of the patients are presented in Table S7, Supporting Information.

To identify the value of the IPS in predicting the response to neoadjuvant immunotherapy, 167 patients with locally advanced GC who received neoadjuvant therapy at the FMUUH from March 2019 to November 2021 were included, of which 63 were excluded owing to the limited area of the tumor region or a lack of biopsy specimens, and 104 were finally included, whose baseline information is shown in Table S8, Supporting Information. All the patients received a chemotherapy regimen based on fluorouracil and nab‐paclitaxel. Of these patients, 52 who received anti‐PD‐1 therapy (camrelizumab) were enrolled in the neoadjuvant immunotherapy cohort. The remaining 52 patients who received chemotherapy alone were classified into the neoadjuvant chemotherapy cohort. The specific treatment regimens and inclusion criteria are detailed in our previous study.^[^ ^27^ ^]^ For efficacy evaluation, the effect of neoadjuvant therapy was evaluated independently by two specialized radiologists following the guidelines of the Response Evaluation Criteria in Solid Tumors (RECIST version 1.1),^[^ ^28^ ^]^ and the final results were determined after a cross‐review of the results. The tumor regression grade (TRG) was determined to evaluate postoperative pathological tissue according to the Becker criteria.^[^ ^29^ ^]^ Pathologic complete response (pCR) was defined as the absence of invasive disease, and total lesions and histologically negative lymph nodes were evaluated. The overall design flowchart of this study is shown in Figure 1B.

The ethics approval number for this research project was 2022KY084 and was obtained from the FMUUH. All seven centers approved this study and all patients signed informed consent forms before tissue collection.

### Consensus Clustering of Immune Phenotypes

The immune cell ratios of GC tissue were quantitated using the CIBERSORT algorithm and LM22 gene signature.^[^ ^30^ ^]^ The package “ConsensuClusterPlus”^[^ ^31^ ^]^ was then applied to perform hierarchical agglomerative clustering (based on the Euclidean distance and Ward's linkage) of the infiltration of five types of immune cells (CD8, memory CD4, and resting memory CD4 T cells; M1 and M2 macrophages), applying an unsupervised clustering (K‐means) approach to identify and classify patients with different immune phenotypes. In this case, a consensus clustering algorithm with 1000 iterations was used to determine the optimal number of clusters (*k* = 3).

### Identification of Immunophenotype‐Associated DEGs

The R package “limma” was used to identify immunophenotype‐associated DEGs.^[^ ^32^ ^]^ To rank the importance of DEGs for the immunophenotype in the five GC datasets (TCGA‐STAD, ACRG/GSE66229, GSE84433, GSE26942, and GSE15459) and to reduce the bias caused by the unequal number of cases in each dataset, a formula was constructed to calculate the weight of each DEG.

(1) $$\begin{array}{ccc} \mathrm{Weight}\left(\mathrm{total}\right) & = & \sum (\mathrm{LogFC}\times \frac{2}{\left(1+\mathrm{adj}\cdot p\;\mathrm{value}\right)}\times \left(\mathrm{AUC}-0.5\right)\\ & & \times \mathrm{Log}2\left(\mathrm{HR}\right)\times \left(\frac{n_{i}}{N}\right)) \end{array}$$

This weight was calculated as the DEGs between IMcluster A and IMcluster B/C. AUC is the area under the ROC curve predicting IM cluster A, HR is the hazard ratio to prognosis in the Cox regression model, *n*_i_ is the number of patients in the individual dataset, and *N* is the sum of cases in the five GC datasets. For each DEG, the weights in each dataset were first obtained and then summed to obtain total weights.

### Immunohistochemistry Staining and Evaluation

Immunohistochemistry was applied on 4 µm‐thick FFPE GC tissues, as described in previous studies. Details of the primary antibodies used are shown in Table S9, Supporting Information. The Motic EasyScan system (Motic, Xiamen, China) and Nikon E200 microscope (Nikon, Tokyo, Japan) were used to acquire images.

For staining of immunophenotype‐associated DEGs, DEGs were quantified using the H‐score

(2) $$\begin{array}{ccc} H-\mathrm{Score} & = & \left(1\;\times \;\mathrm{Weak}\;\mathrm{Stain}\;\%)\;+\;(2\;\times \;\mathrm{Medium}\;\mathrm{Stain}\;\%\right)\\ & & +(3\;\times \;\mathrm{Strong}\;\mathrm{Stain}\;\%) \end{array}$$

The immunohistochemical scoring criteria (WARS, UBE2L6, GZMB, BATF2, and LAG‐3) are shown in Figure S11A, Supporting Information.

Four mismatch repair proteins (MSH2, MSH6, MLH1, and PMS2) were employed to determine MSI status. The scoring criteria (Figure S11B, Supporting Information) were at least one missing mismatch repair gene‐related protein, interpreted as dMMR, manifested as MSI‐H; no missing mismatch repair gene‐related protein was interpreted as proficient MMR, manifested as MSI‐L/MSS. EBV status was measured using in situ hybridization using an EBER probe (ISH‐6021, ZSGB‐BIO, China; Figure S11B, Supporting Information).

To assess the infiltration of immune cells, five representative 200× fields of view were acquired in the CT and the IM of each GC tissue to calculate the positive cell density (Figure S11C, Supporting Information).

(3) $$\mathrm{Density}\;=\;\frac{C_{P1}+\;C_{P2}+\;C_{P3}+\;C_{P4}+\;C_{P5}}{{\mathrm{Area}}_{1}+{\;\mathrm{Area}}_{2}+{\;\mathrm{Area}}_{3}+{\;\mathrm{Area}}_{4}+{\;\mathrm{Area}}_{5}}$$

*C*_P*x*_ is the total number of cells stained positive in this field, Area_*x*_ is the area of this field. The positive cell count was assisted by the measurement plugin of Image Pro Plus software (version 6.0, Media Cybernetics, USA), which was also used to determine the area of the tumor region. The inflamed, excluded, and desert phenotypes were determined based on immunohistochemical staining slides for CD8^+^, and these three immunophenotypes were classified based on the characteristics reported in previous studies. PD‐L1 expression was measured using the CPS

(4) $$\mathrm{CPS}\;=\;\frac{\mathrm{total}\;\mathrm{number}\;\mathrm{of}\;\mathrm{positive}\;\mathrm{stain}\;\mathrm{cells}\;\mathrm{for}\;\mathrm{PD}-L1}{\mathrm{total}\;\mathrm{number}\;\mathrm{of}\;\mathrm{viable}\;\mathrm{tumor}\;\mathrm{cells}}\times 100$$

Cells positive for PD‐L1 include PD‐L1‐expressing tumor cells, lymphocytes, and macrophages.

Two senior pathologists, blinded to the clinicopathological features and prognosis of the patients, independently scored all samples. For the assessment of immunophenotype‐related DEGs, 74.7% of the samples were scored in complete agreement and 23.8% differed by 10% or less. The two pathologists were in complete agreement with the determination of the MSI and EBV status. When the difference between the scores of the two independent pathologists was within 10%, the average of the two was considered; when the difference was greater than 10%, a third pathologist reviewed the results and selected one of the scores from the first two pathologists or the three pathologists reached consensus.

### Construction of the IPS

The LASSO Cox and randomSurvivalForest algorithms were used to identify immunophenotype‐associated DEGs associated with GC prognosis. In the LASSO Cox model, Lambda (−4.2503), selected using the least deviation likelihood ratio, failed to exclude any of the eight genes. RandomSurvivalForest was performed simultaneously. Interestingly, the five genes that were included when considering the importance of 0.03 as the threshold were the same as those included in the LASSO analysis when the lambda was −2.2966. The ROC curves corresponding to the two lambdas were compared, and it was found that the AUC decreased by only 0.01, after excluding three genes, which was acceptable for improving the simplicity of using the model. Thus, these five genes (GZMB, WARS, LAG‐3, BATF2, and UBE2L6) were included in the final model construction. To reduce bias in model construction, specimens from the remaining 253 patients with GC in the Training Cohort were also stained for the five genes. Next, multivariate Cox proportional hazards regression with a stepwise procedure was performed to obtain predictive models based on immunophenotype‐related DEGs, and risk scores for each patient were obtained using the R package “survival.” Because the scoring model was constructed based on the immunophenotype, the risk score was named the IPS. The “ggriskee” package was used to determine the Youden index of the risk score and classify patients into IPS^Low^ and IPS^High^ groups depending on this score.

### Multiplex Immunohistochemistry Staining

An Opal 7‐color Kit (NEL871001KT, Akoya Bioscience, USA) was used to perform multiplex immunohistochemical staining to identify M1 and M2 macrophages (CD68/CD163/CD206/INOS). Effector T cells (Teffs; GZMB/CD8) were characterized separately in two separate panels using an Opal 4‐color Kit (NEL840001KT, Akoya Bioscience, USA). Labeling of multi‐cytokeratin using panCK (ab7753, Abcam, UK) in all panels was used to segment the tumor nest and stroma or determine the IM. The nuclei of all cells were stained with DAPI (D9542; Sigma‐Aldrich, USA). The details of the primary antibodies are presented in Table S9, Supporting Information.

A Mantra System (PerkinElmer, USA) was used to capture multispectral panoramic images after staining. The scanned slides were then analyzed using the InForm software (PerkinElmer, USA) to obtain quantitative data on the region of interest (ROI). InForm can accurately count positive cells and identify them by setting reasonable thresholds, which allowed for the computation of the density and ratio of the target cells in the ROI. It also allowed automated segmentation of the tumor nest (panCK^+^) and stroma (panCK^−^) to collect quantitative data from different ROIs.

### Identification of IPS Applicability on Gastroscopic Biopsy Specimens

To determine whether the IPS established based on postoperative pathology specimens can be applied to smaller gastroscopic biopsy specimens, 112 patients with GC from the Discovery Cohort were enrolled, their biopsy tissues were paired with postoperative tissues, and their respective IPS were assessed (Figure S9A, Supporting Information). None of the 112 patients received neoadjuvant therapy to ensure that the intrinsic characteristics of biopsy and postoperative tissues were consistent. The area of the tumor region was measured, and the IPS in the biopsy tissue was evaluated. Interestingly, when biopsy tissue with an assessable tumor area of <0.16 mm^2^ was excluded, the IPS of the biopsy tissue (IPS_b_) had the highest correlation with IPS of postoperative tissue (IPS_p_), and IPS_b_ had the highest AUC value for predicting IPS_p_ (Figure S9B, Supporting Information). Although elevated exclusion criteria may result in better accuracy of IPS assessment, a sample size that is too large may be lost. Finally, 52 of 83 patients receiving neoadjuvant ICI therapy in combination with chemotherapy were included in the study (31 were excluded; 22 due to an assessable tumor area of <0.16 mm^2^ and 9 due to a lack of biopsy specimen).

### Statistical Analysis

The LASSO regression model was analyzed using the R package “glmnet,” while randomSurvivalForest was constructed by the practical R package “randomForestSRC.” The Mann–Whitney U‐test was used to compare two groups of non‐normally distributed continuous variables, while the Kruskal–Wallis test was used to perform multiple comparisons. Categorical variables of clinicopathological characteristics were compared using the *χ* ^2^ test or Fisher's exact test. Nonparametric correlation analyses were performed using Spearman's test. Kaplan–Meier survival analysis with a log‐rank test was used to estimate OS. The univariate Cox proportional hazard regression model confirmed the association between the relevant clinicopathological variables and OS. Next, multivariate Cox regressions were included to analyze statistically significant indicators (*p* ≤ 0.05) in the univariate analysis (training cohort: IPS, tumor size, degree of differentiation, pTNM stage, CEA, and CA19‐9; Central China cohort: IPS, degree of differentiation, pTNM stage; North China cohort: IPS and pTNM stage; South China cohort: IPS, degree of differentiation, and pTNM stage). Univariate and multivariate logistic regression analyses were used to determine the association between the variables and response to immunotherapy.

## Conflict of Interest

The authors declare no conflict of interest.

## Author Contributions

J.‐B.W., Q.‐Z.Q., Q.‐L.Z., Y.‐J.Z., Y.X., T.Z., S.‐H.W., Q.W., and Q.‐W.J. contributed equally to this work. J.‐B.W. and Q.‐Z.Q. conceived the study and drafted the manuscript. C.‐M.H. and C.‐H.Z. helped critically revise the manuscript for important intellectual content. Q.‐Z.Q., Q.‐L.Z., and Y.‐H.Y. performed the research. Y.‐H.Y., Y.‐J.Z., Y.X., T.Z., S.‐H.W., Q.W., and Q.‐W.J. provided tissue samples from GC patients. P.L., J.‐W.X., J.‐X.L., J.L., Q.‐Y.C., and L.‐L.C. helped collect data and design the study.

## Supporting information

Supporting Information

## Data Availability

The data that support the findings of this study are available from the corresponding author upon reasonable request.
