# Supplementary material for: Tumor Immunophenotyping‐Derived Signature Identifies Prognosis and Neoadjuvant Immunotherapeutic Responsiveness in Gastric Cancer
Source: Adv Sci (Weinh). 2023 Mar 30;10(15):2207417. doi: 10.1002/advs.202207417 (PMC10214263; doi:10.1002/advs.202207417)
Supplement: Supplementary file 1 — Supporting Information [file ADVS-10-2207417-s001.pdf]

ADVANCED SCIENCE
Open Access

Supporting Information

for *Adv. Sci.*, DOI 10.1002/advs.202207417

Tumor Immunophenotyping-Derived Signature Identifies Prognosis and Neoadjuvant Immunotherapeutic Responsiveness in Gastric Cancer

Jia-Bin Wang, Qing-Zhu Qiu, Qiao-Ling Zheng, Ya-Jun Zhao, Yu Xu, Tao Zhang, Shuan-Hu Wang, Quan Wang, Qin-Wen Jin, Yin-Hua Ye, Ping Li, Jian-Wei Xie, Jian-Xian Lin, Jun Lu, Qi-Yue Chen, Long-Long Cao, Ying-Hong Yang, Chao-Hui Zheng* and Chang-Ming Huang**

WILEY-VCH

Supporting Information

Tumor immunophenotyping-derived signature identifies prognosis and neoadjuvant immunotherapeutic responsiveness in gastric cancer

Jia-Bin Wang†, *Qing-Zhu Qiu*†, *Qiao-Ling Zheng*†, *Ya-Jun Zhao*†, *Yu Xu*†, *Tao Zhang*†, *Shuan-Hu Wang*†, *Quan Wang*†, *Qin-Wen Jin*†, *Yin-Hua Ye, Ping Li, Jian-Wei Xie, Jian-Xian Lin, Jun Lu, Qi-Yue Chen, Long-Long Cao, Ying-Hong Yang*, Chao-Hui Zheng*, Chang-Ming Huang**

† **Contributed equally**

*** Corresponding authors:** Chang-Ming Huang (***hcmlr2002@163.com***)

Chao-Hui Zheng (***wwkzch@163.com***)

Ying-Hong Yang (***yyh1555@163.com***)

Supplementary Methods

Functional and pathway enrichment analysis

For gene set functional enrichment analysis, we performed enrichment analysis using GO annotations of genes from the R package ‘org.Hs.eg.db’ (version 3.14.0) as background, mapped genes to the background set, and used the R package ‘clusterProfiler’ (version 4.22) to obtain gene set enrichment results. GSEA analysis was performed using software from the GSEA (http://software.broadinstitute.org/gsea/index.jsp) website (version 4.1.0, Broad Institute). Significant enrichment conditions were NES > 1 or < -1 and FDR-q-value < 0.05.

Figure S1

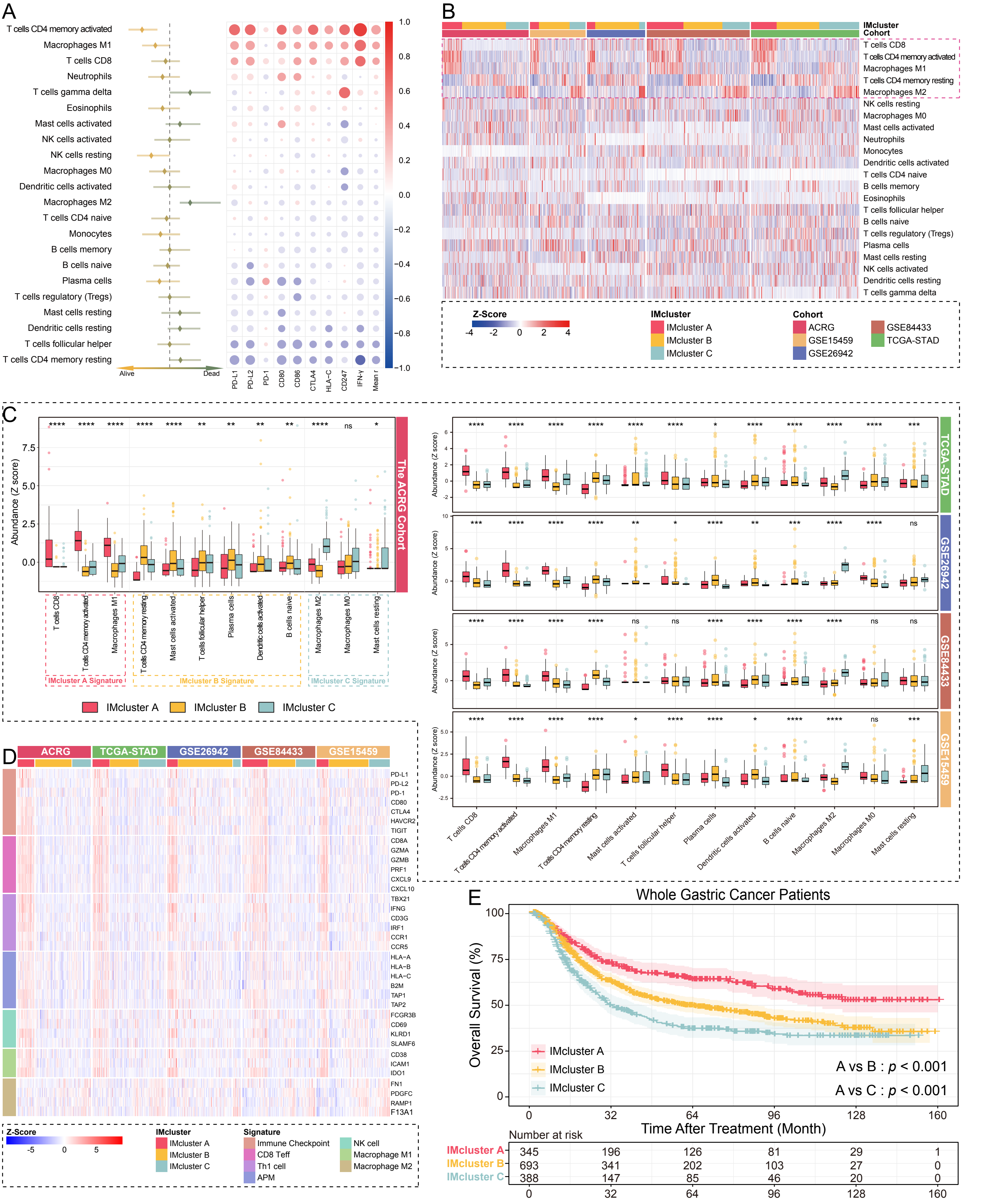

Figure S1. Characterization of sub-clusters of immunophenotypes in gastric cancer (**A**) The left panel presents the prognostic value of the TME cell types, and the right panel presents the correlation matrix of the TME cell types with immune checkpoints and related transcripts. (**B**) Unsupervised clustering in five publicly available GC datasets. (**C**) Expression of the TME cells in three immunophenotypic clusters (IMclusters). *$p < 0.05$; **$p < 0.01$; ***$p < 0.001$; ****$p < 0.0001$, Kruskal–Wallis test. The thick line shows the median value. The ACRG Cohort (IMcluster A: n = 70; IMcluster B: n = 152; IMcluster C: n = 78), TCGA-STAD (IMcluster A: n = 88; IMcluster B: n = 148; IMcluster C: n = 139), GSE26942 (IMcluster A: n = 29; IMcluster B: n = 152; IMcluster C: n = 21), GSE84433 (IMcluster A: n = 127; IMcluster B: n = 135; IMcluster C: n = 95), GSE15459 (IMcluster A: n = 31; IMcluster B: n = 106; IMcluster C: n = 55). The bottom and top of the boxes are the 25th and 75th percentile (interquartile range) and extend through the whiskers to 1.5 times the interquartile range. (**D**) Heatmap illustrating the expression profile of immune-related transcripts according to IMclusters. (**E**) Kaplan–Meier survival curves for overall survival (OS) of the GC patients in the five datasets demonstrate prognostic characteristics of the three IMclusters (log-rank test).

Figure S2

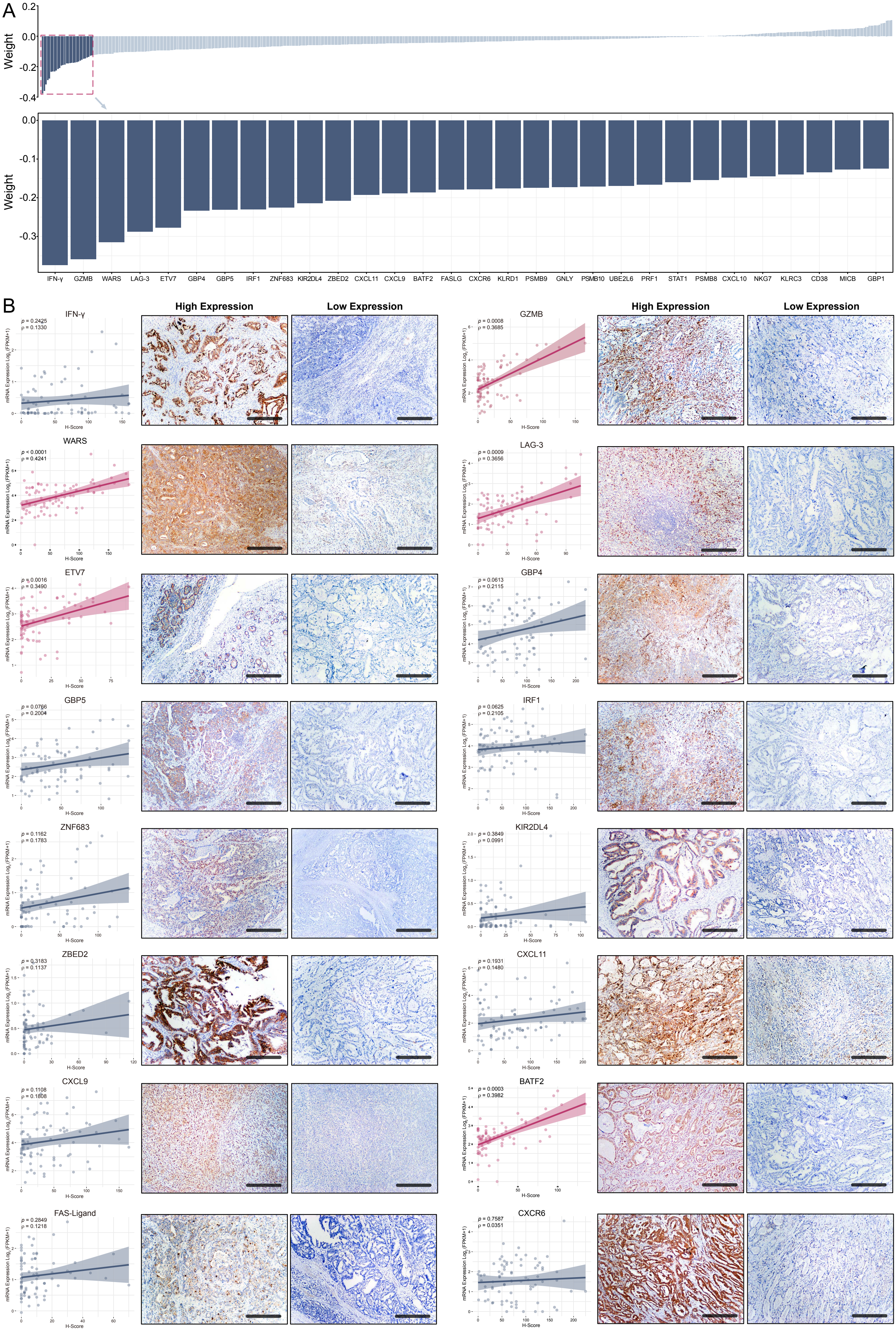
A
Weight
0.2
0.0
-0.2
-0.4
0.0
-0.1
-0.2
-0.3
IFN-γ
GZMB
WARS
LAG-3
ETV7
GBP4
GBP5
IRF1
ZNF683
KIR2DL4
ZBED2
CXCL11
CXCL9
BATF2
FASLG
CXCR6
KLRD1
PSMB9
GNLY
PSMB10
UBE2L6
PRF1
STAT1
PSMB8
CXCL10
NKG7
KLRC3
CD38
MICB
GBP1
B
High Expression
Low Expression
IFN-γ
p = 0.2425
ρ = 0.1330
GZMB
p = 0.0008
ρ = 0.3685
WARS
p < 0.0001
ρ = 0.4241
LAG-3
p = 0.0009
ρ = 0.3656
ETV7
p = 0.0016
ρ = 0.3490
GBP4
p = 0.0613
ρ = 0.2115
GBP5
p = 0.0766
ρ = 0.2004
IRF1
p = 0.0625
ρ = 0.2105
ZNF683
p = 0.1162
ρ = 0.1783
KIR2DL4
p = 0.3849
ρ = 0.0991
ZBED2
p = 0.3183
ρ = 0.1137
CXCL11
p = 0.1931
ρ = 0.1480
CXCL9
p = 0.1108
ρ = 0.1808
BATF2
p = 0.0003
ρ = 0.3982
FAS-Ligand
p = 0.2849
ρ = 0.1218
CXCR6
p = 0.7587
ρ = 0.0351
mRNA Expression Log2 (FPKM+1)
H-Score

Figure S2 (continued)

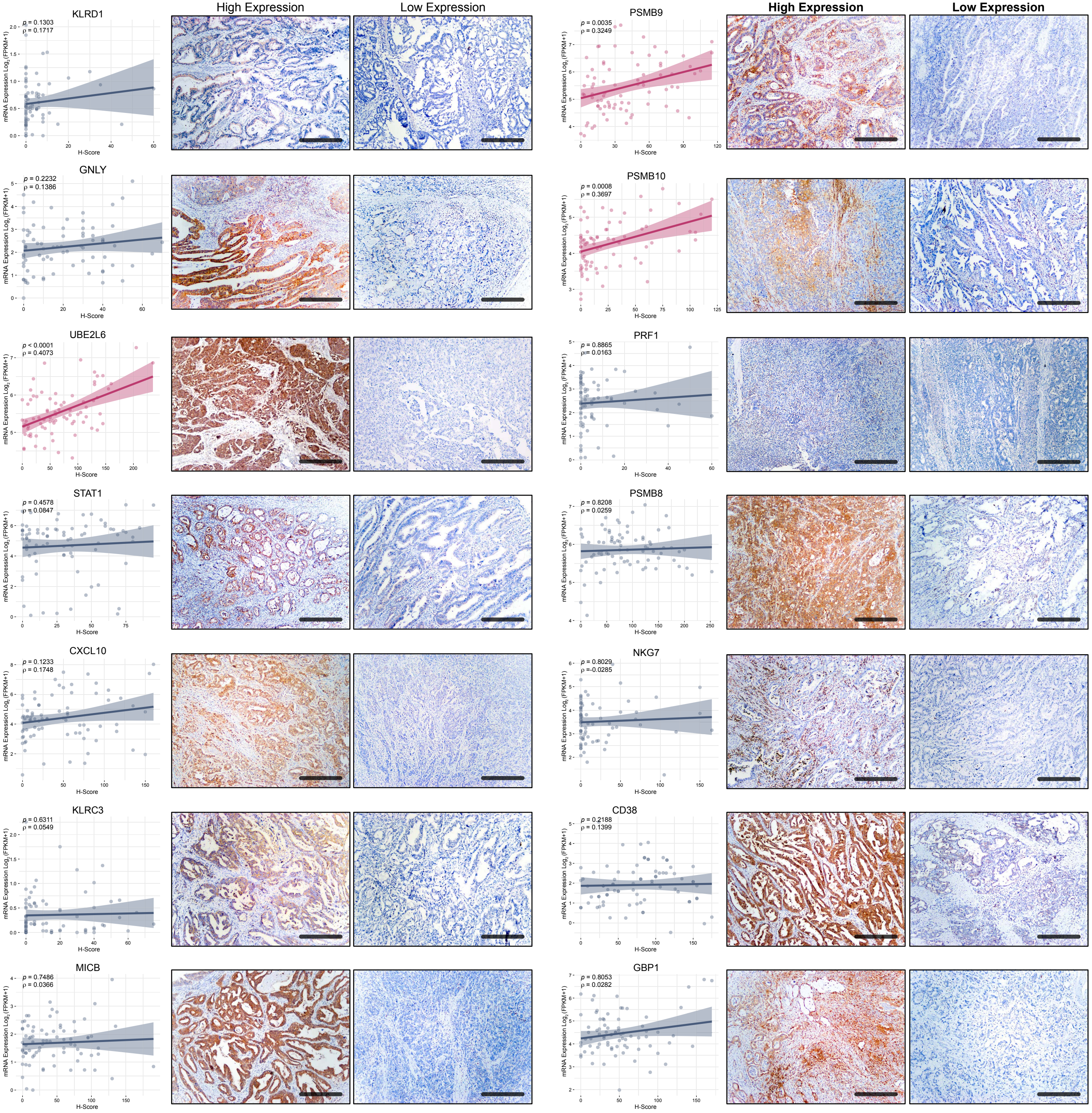

Figure S2. Screening for translational-transcriptional coherence of core molecules regulating IMcluster A. (**A**) Top 30 weighted transcripts in immune phenotype-associated differentially expressed genes (DEGs). (**B**) Correlation between transcriptional expression levels (mRNA , FPKM) and translational expression levels (immunohistochemistry, H-score) of the top 30 weighted genes in the FMUUH-RNA_Seq Cohort (n = 79). Red represents genes with a correlation coefficient *p* ≥ 0.3, indigo represents genes with a correlation coefficient *p* < 0.3, Spearman's test.

Figure S3

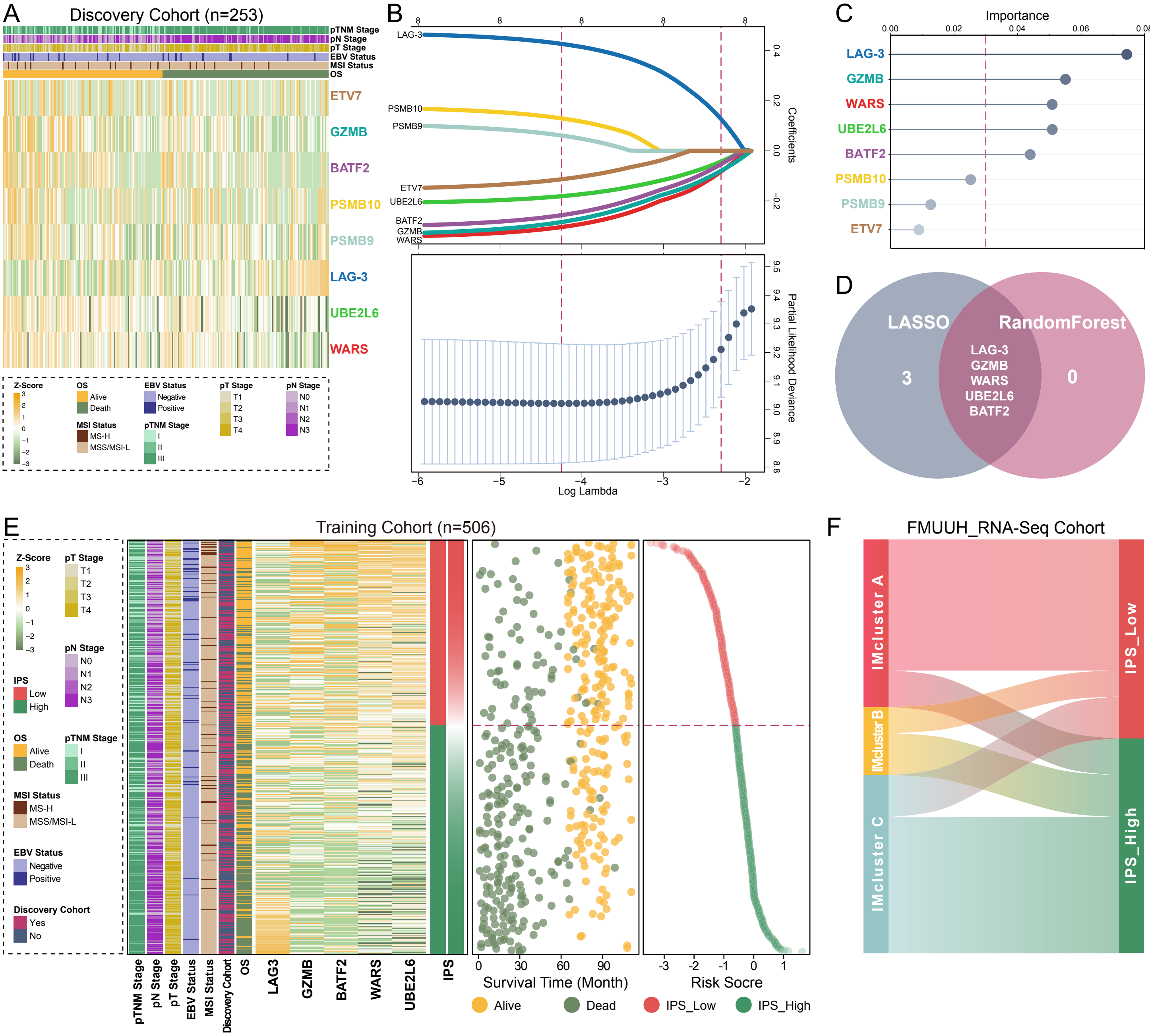

Figure S3. Construction of immunophenotype-based signatures for pathological tissues. (**A**) Immunohistochemical scores (H-score) for eight indicators with consistent transcriptional and translational levels in the Discovery Cohort (n = 253). (**B**) LASSO Cox regression to determine the optimal Lambda and the corresponding coefficients of eight indicators. (**C**) randomForest demonstrates the importance of the eight indicators for prognosis. (**D**) The screening results of LASSO Cox and randomForest are presented in the Venn diagram. (**E**) A stepwise multifactorial Cox proportional regression risk model was applied to obtain a risk score for each patient with GC in the Training Cohort (n = 506), and patients were classified into low- and high-risk groups according to an optimal cut-off point (−0.606). (**F**) The Sankey diagram exhibits the correspondence between IMcluster and immunophenotype score (IPS) in the FMUUH-RNA_Seq Cohort (n = 79).

Figure S4

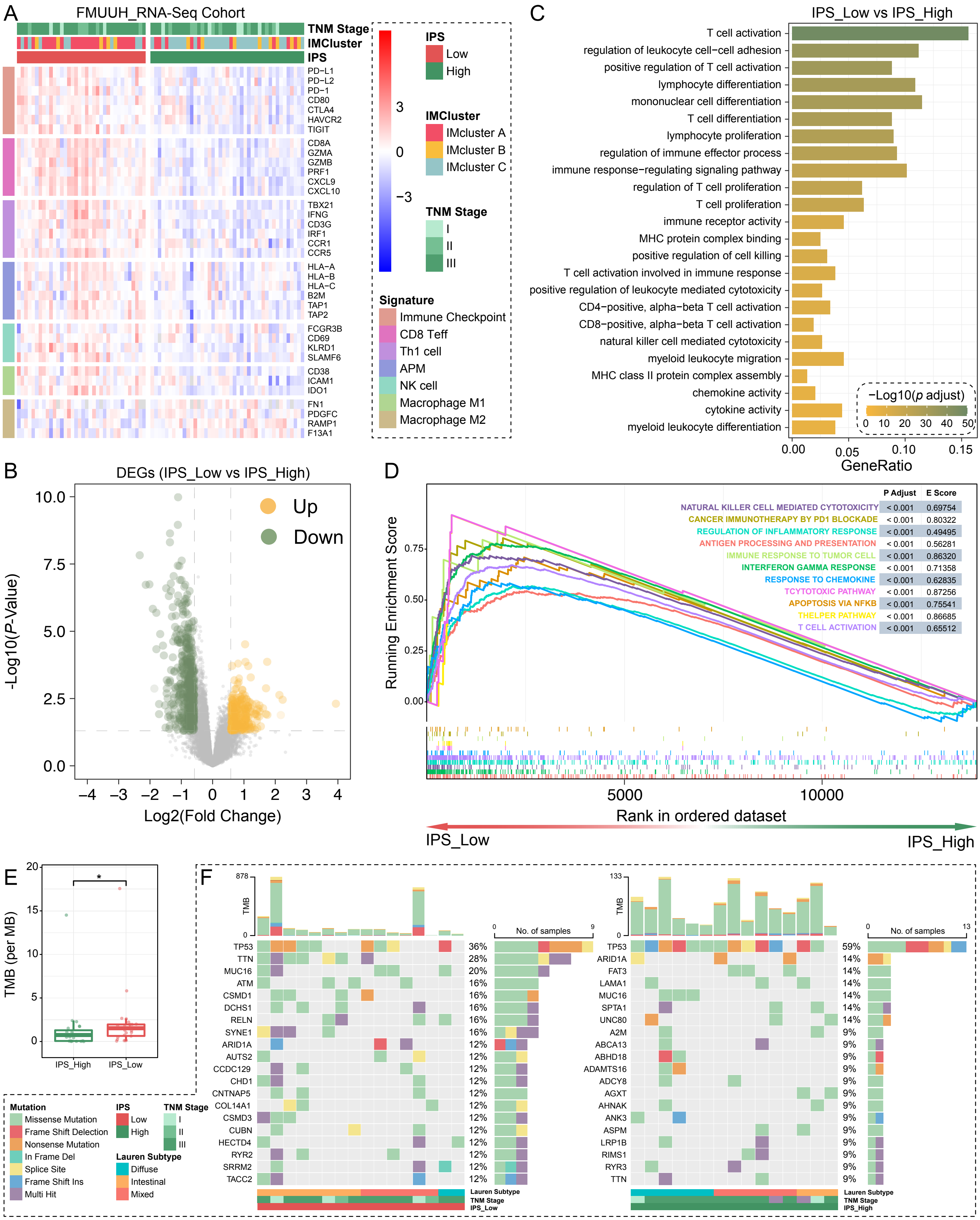

Figure S4. Characterization of the transcriptome and genome of the IPS. (**A**) The heatmap displays the expression profiles of immune-related transcripts in IPS^{Low} and IPS^{High} in the FMUUH-RNA_Seq Cohort (n = 79). (**B**) Differentially expressed genes (DEGs) of IPS^{Low} over IPS^{High}. (**C**) GO enrichment analysis of DEGs in IPS^{Low} versus IPS^{High}. (**D**) The enrichment plot demonstrates the immune-related hallmark gene sets. All transcripts were ranked by log2 (fold change) between IPS^{Low} and IPS^{High}. (**E**) Forty-seven patients with GC with whole-exome sequencing (WES) of the FMUUH-RNA_Seq Cohort, comparing the tumor mutation burden (TMB) in IPS^{Low} (n = 22) versus IPS^{High} (n = 25). *$p < 0.05$, Mann-Witney U test. The thick line shows the median value. The bottom and top of the boxes are the 25th and 75th percentile (interquartile range) and extend through the whiskers to 1.5 times the interquartile range. (**F**) The oncoPrint illustrates the mutation landscape of IPS^{Low} (left panel) and IPS^{High} (right panel) patients with GC from the FMUUH-RNA_Seq Cohort.

Figure S5

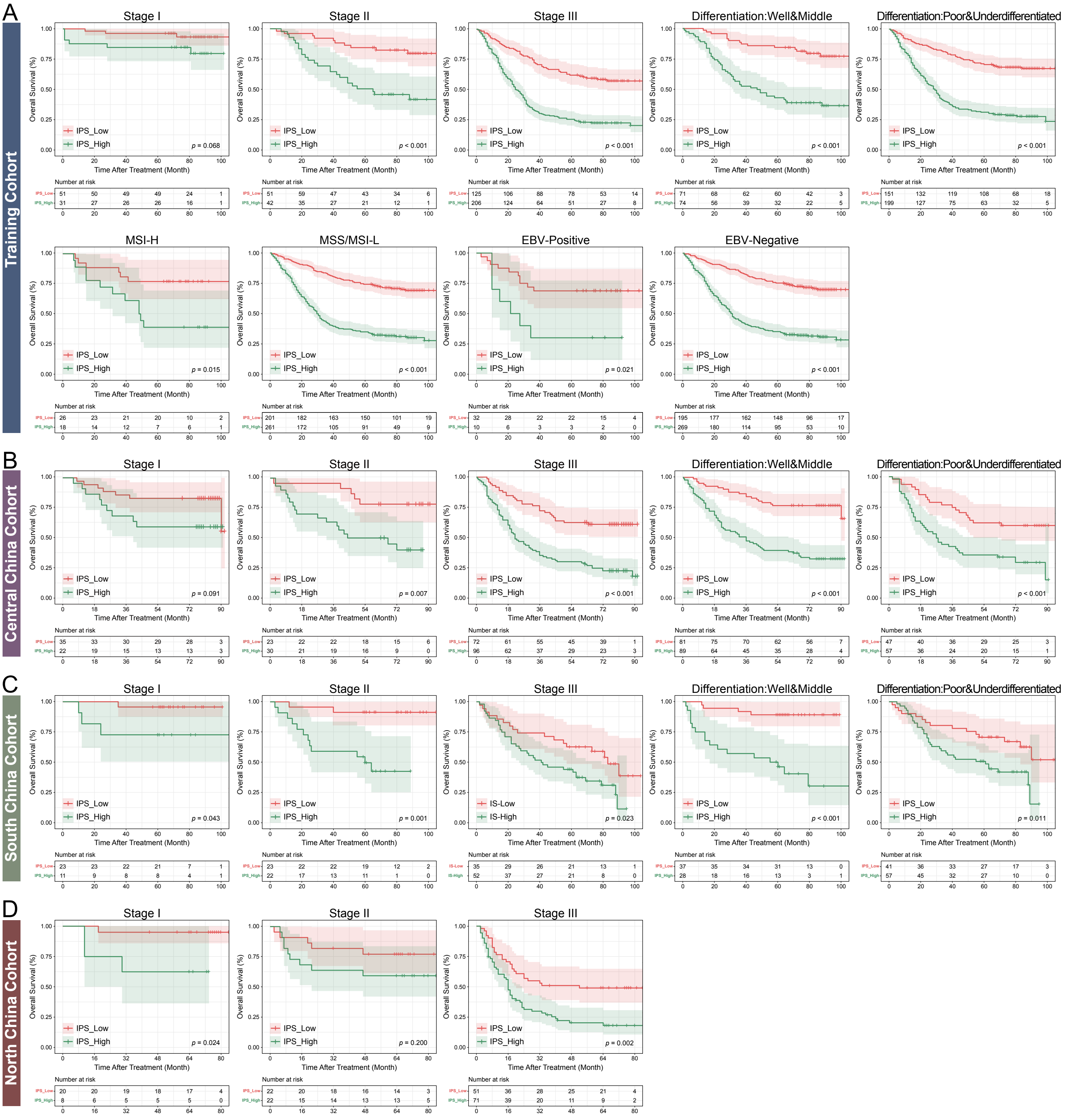

Figure S5. Kaplan–Meier survival curves demonstrate the results of stratification analysis according to clinico-pathological characteristics in four individual cohorts. (**A**) The Training Cohort (pTNM Stage, differentiation, MSI status, and EBV status). (**B**) The Central China Cohort (pTNM Stage and differentiation). (**C**) The South China Cohort (pTNM Stage and differentiation). (**D**) The North China Cohort (pTNM Stage). Comparison of overall survival by log-rank test.

Figure S6

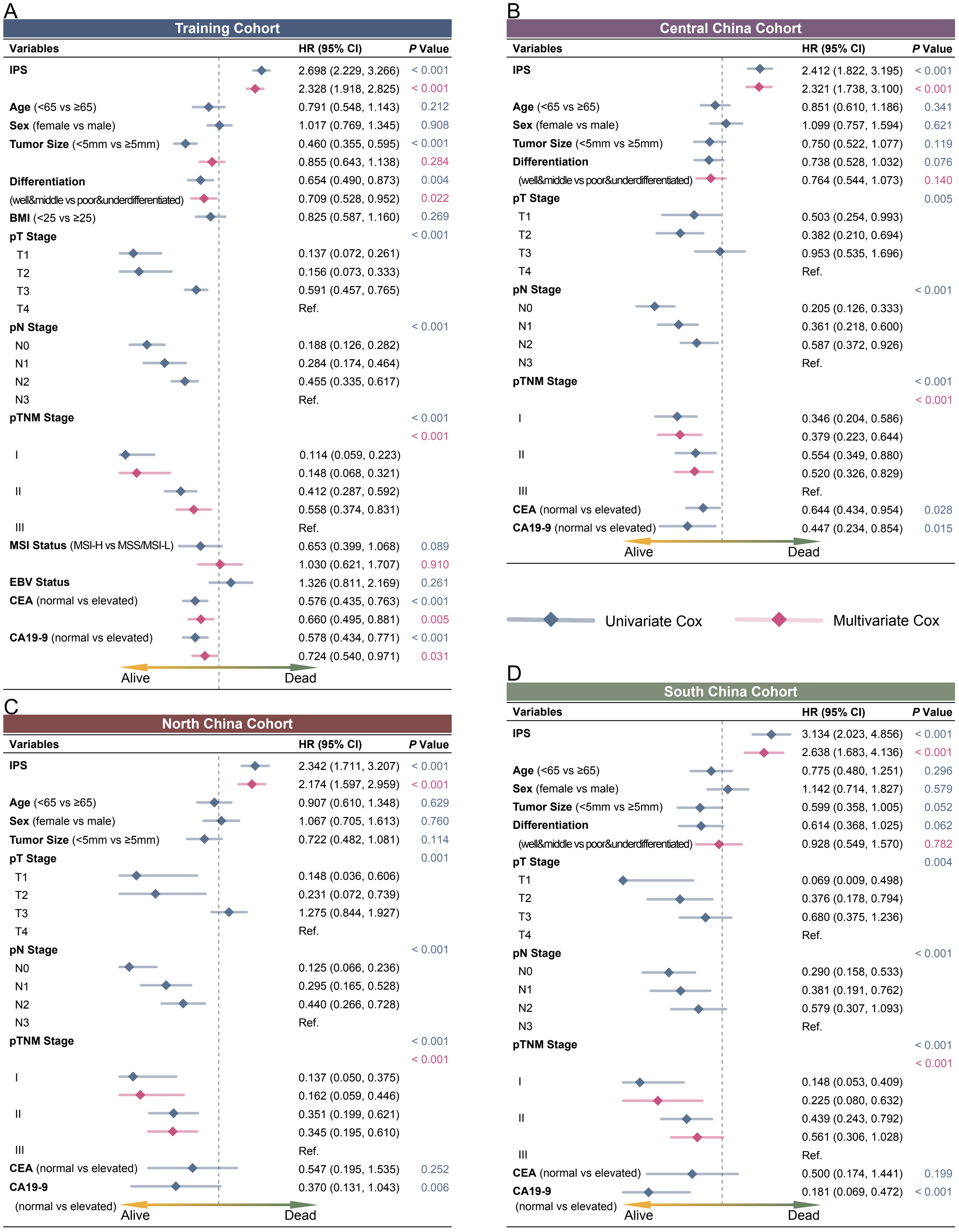

Figure S6. Univariate and multivariate Cox regression analysis indicates that the IPS is an independent and robust biomarker for predicting prognosis. (**A**) Training Cohort. (**B**) Central China Cohort. (**C**) North China Cohort. (**D**) South China Cohort. Variables with statistical significance in the univariate analysis were integrated into the multivariate analysis. The dotted line represents the hazard ratio (HR) = 1. Training Cohort: n = 506, Central China Cohort: n = 178, North China Cohort: n = 194, South China Cohort: n = 166. The indigo symbols and words represent univariate Cox analysis, while red represents multivariate Cox analysis.

Figure S7

WILEY-VCH

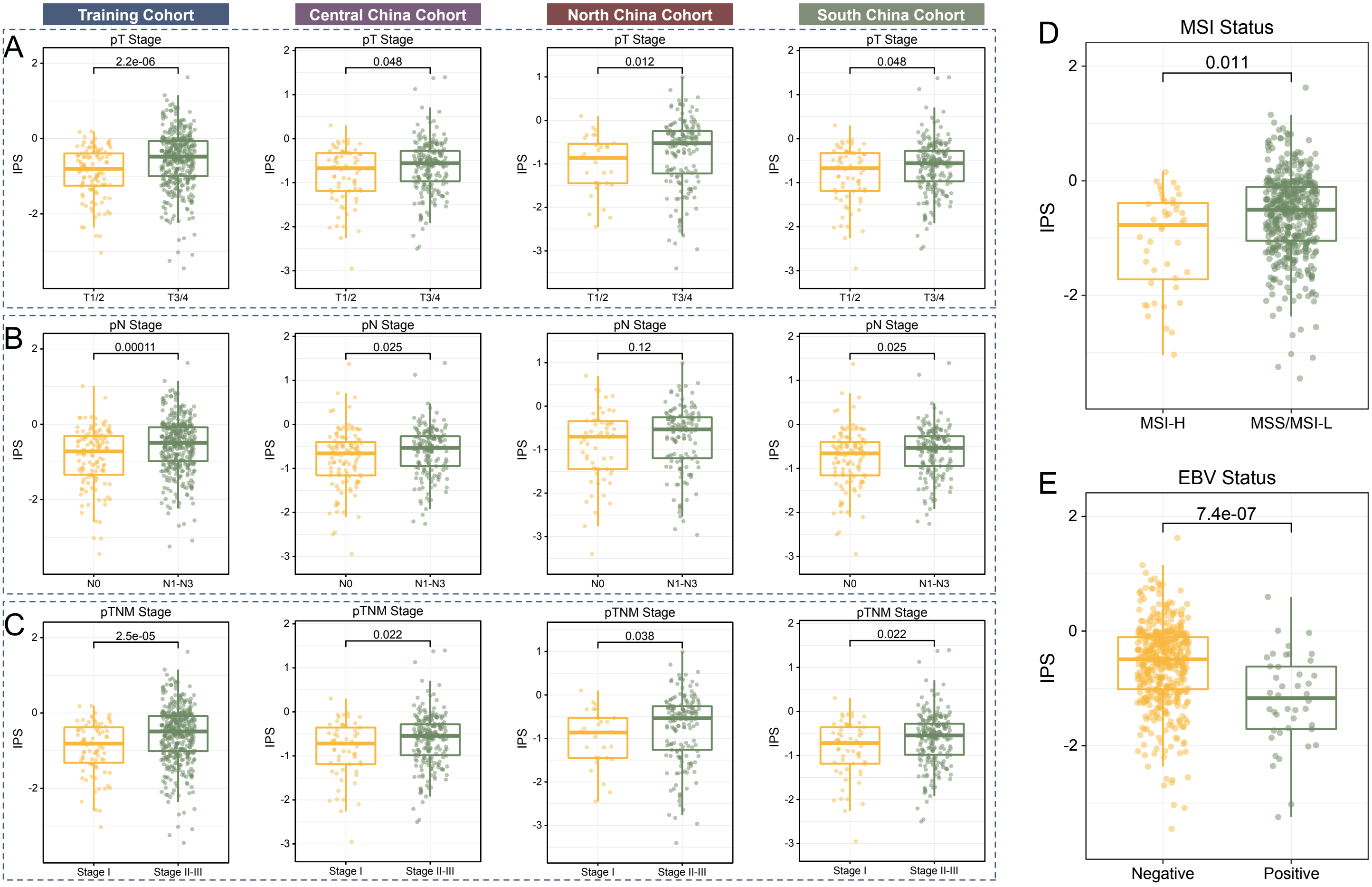

Figure S7. IPS is associated with tumor stage, MSI, and EBV status. (**A**) Comparison of IPS between the pT1/pT2 and pT3/pT4 stage in the four cohorts. (**B**) Comparison of the IPS between the pN0 and pN1/pN3 stage in the four cohorts. (**C**) Comparison of the IPS between Stage I and II–III in the four cohorts. Training Cohort: n = 506, Central China Cohort: n = 178, North China Cohort: n = 194, South China Cohort: n = 166. The detailed number of patients is presented in **Tables S5** and **S6**. (**D**) Comparison of the IPS between MSI-H (n = 44) and MSS/MSI-L (n = 462) in the Training Cohort. (**E**) Comparison of the IPS between EBV-positive (n = 42) and EBV-negative (n = 464) in the Training Cohort. Mann-Whitney U test. The thick line shows the median value. The bottom and top of the boxes are the 25th and 75th percentile (interquartile range) and extend through the whiskers to 1.5 times the interquartile range.

Figure S8

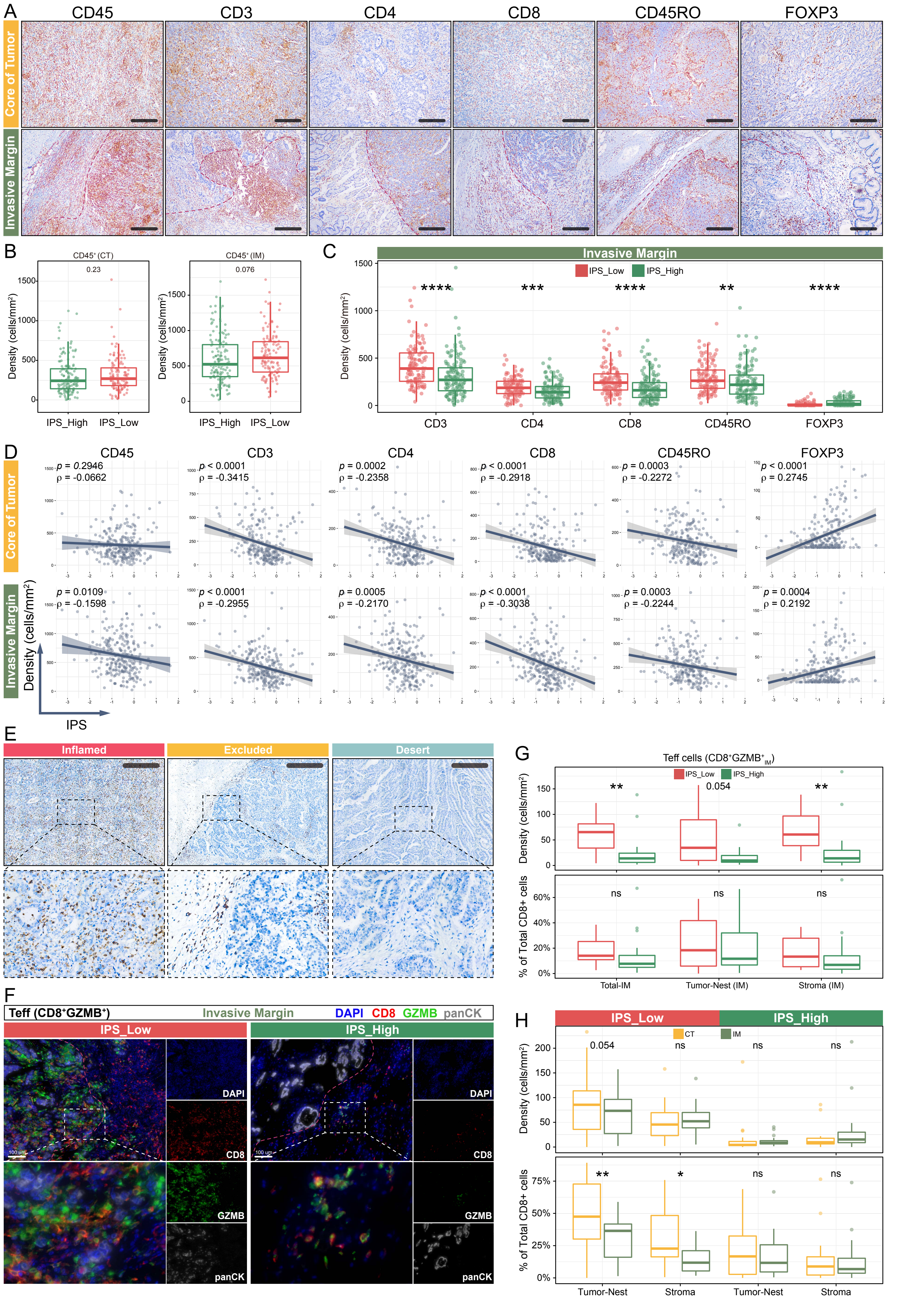

Figure S8. The IPS-specific landscape of the tumor immune microenvironment. (**A**) Immunohistochemical staining of immune cells (CD45⁺, CD3⁺, CD4⁺, CD8⁺, CD45RO⁺, and FOXP3⁺). The upper panel is the scene of the core of tumor (CT) and the lower panel is the scene of the invasive margin (IM). Scale bar = 200 μm. (**B**) Comparison of total leukocyte (CD45⁺) infiltration in IPS^{Low} versus IPS^{High}. Mann–Whitney U-test. (**C**) Comparison of immune infiltration in the IM (CD3⁺, CD4⁺, CD8⁺, CD45RO⁺, and FOXP3⁺) between the IPS^{Low} and IPS^{High} in the Discovery Cohort (n = 253, IPS^{Low} = 115, IPS^{High} = 138). ***$p < 0.001$; ****$p < 0.0001$, Mann–Whitney U-test. (**D**) Correlation of IPS with immune infiltration (CD45⁺, CD3⁺, CD4⁺, CD8⁺, CD45RO⁺, and FOXP3⁺) in CT and IM (n = 253). Spearman's test. (**E**) Immunohistochemical staining slides for CD8A were evaluated for Inflamed, Excluded, and Desert immunophenotypes. Scale bar = 200 μm. (**F**) Multiplexed immunohistochemical staining was used to visualize the effector T cells (Teffs; GZMB⁺CD8⁺) in the IM of IPS^{Low} vs IPS^{High}, and panCK⁺ was used to segment the tumor-nest and stroma (CD8-red, GZMB-green, panCK-grey, and DAPI-blue; n = 31; scale bar = 100 μm). (**G** and **H**) Comparison of the density and ratio (to total CD8⁺ cells) of Teffs between IPS^{Low} and IPS^{High} (Mann–Whitney U-test), and the distribution characteristics of Teffs in different locations of the tumor-nest and stroma (Wilcoxon matched-pairs signed rank test). IPS^{Low}: n = 15, IPS^{High}: n = 16; *$p < 0.05$; **$p < 0.01$. In all box plots of this Figure, the thick line shows the median value. The bottom and top of the boxes are the 25th and 75th percentile (interquartile range) and extend through the whiskers to 1.5 times the interquartile range.

Figure S9

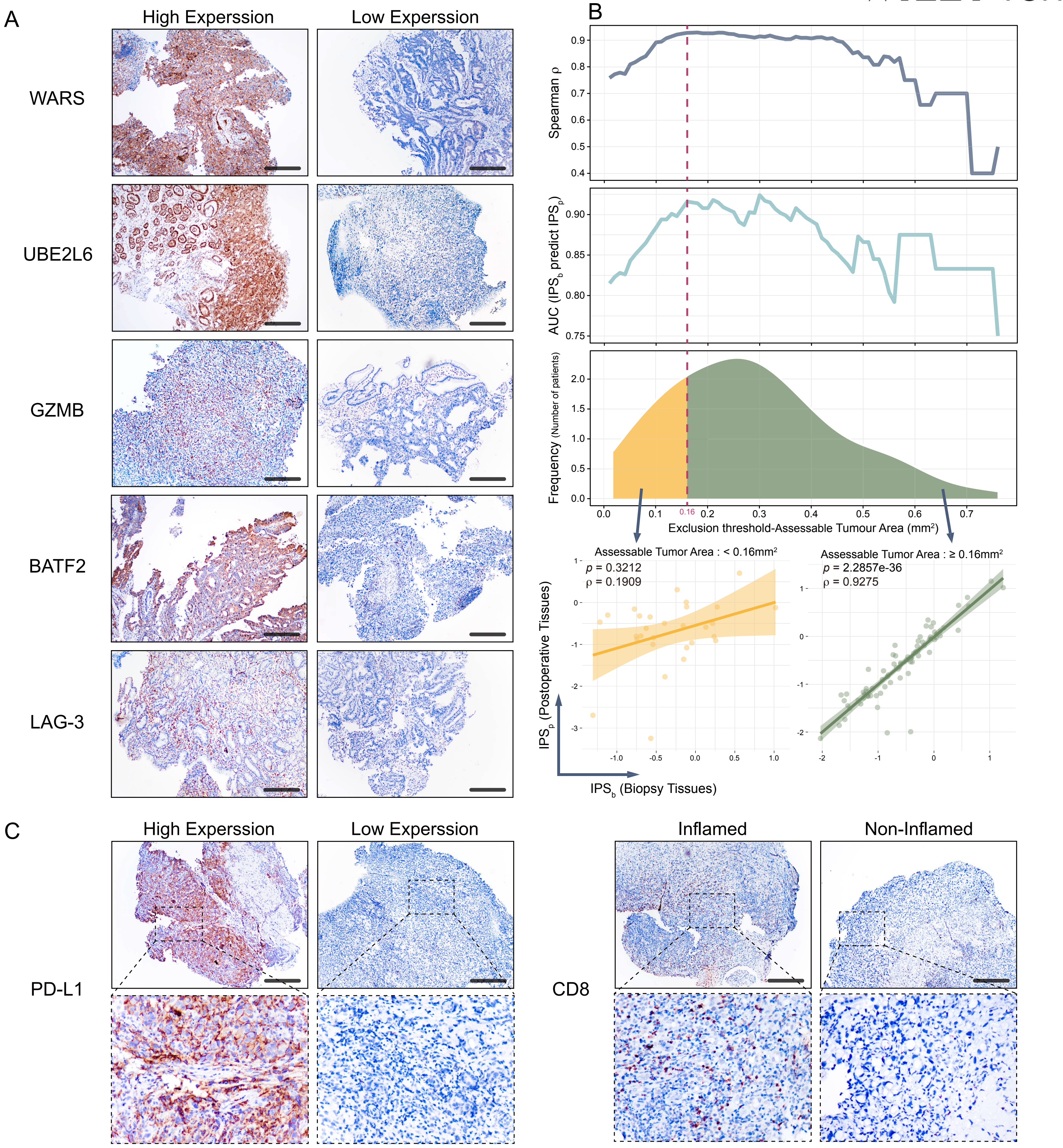

Figure S9. Biopsy specimens obtained by gastroscopy are applicable for evaluation of the IPS. (**A**) Immunohistochemical staining of WARS, UBE2L6, GZMB, BATF2, and LAG-3 was performed on biopsy specimens obtained by gastroscopy. Scale bar = 200 μm. (**B**) Identification of the consistency of the IPS evaluated through gastroscopically obtained biopsy specimens (IPS_b) with the IPS evaluated by postoperative specimens (IPS_p) in the 112 patients from the Discovery Cohort. The horizontal axis is the exclusion threshold for the assessable tumor area (ATA), indicating that specimens with an ATA smaller than the threshold were excluded from the analysis corresponding to the vertical axis. The vertical axis of the upper panel represents the correlation analysis between IPS_b and IPS_p in the included specimens (specimens with an ATA larger than the corresponding horizontal coordinate). The vertical axis in the middle panel indicates the accuracy of IPS_b in predicting IPS_p in the included sample (specimens with an ATA larger than the corresponding horizontal coordinate). The vertical axis in the lower panel reveals the frequency of the number of specimens in this ATA interval. Yellow dots represent the correlation analysis between IPSb and IPSp for specimens with an ATA < 0.16 mm^2 (n = 29), and green dots represent the correlation analysis between IPS_b and IPS_p for specimens with an ATA > 0.16 mm^2 (n = 83). (**C**) Immunohistochemical staining of PD-L1 and CD8A was performed on biopsy specimens obtained by gastroscopy. Scale bar = 200 μm.

Figure S10

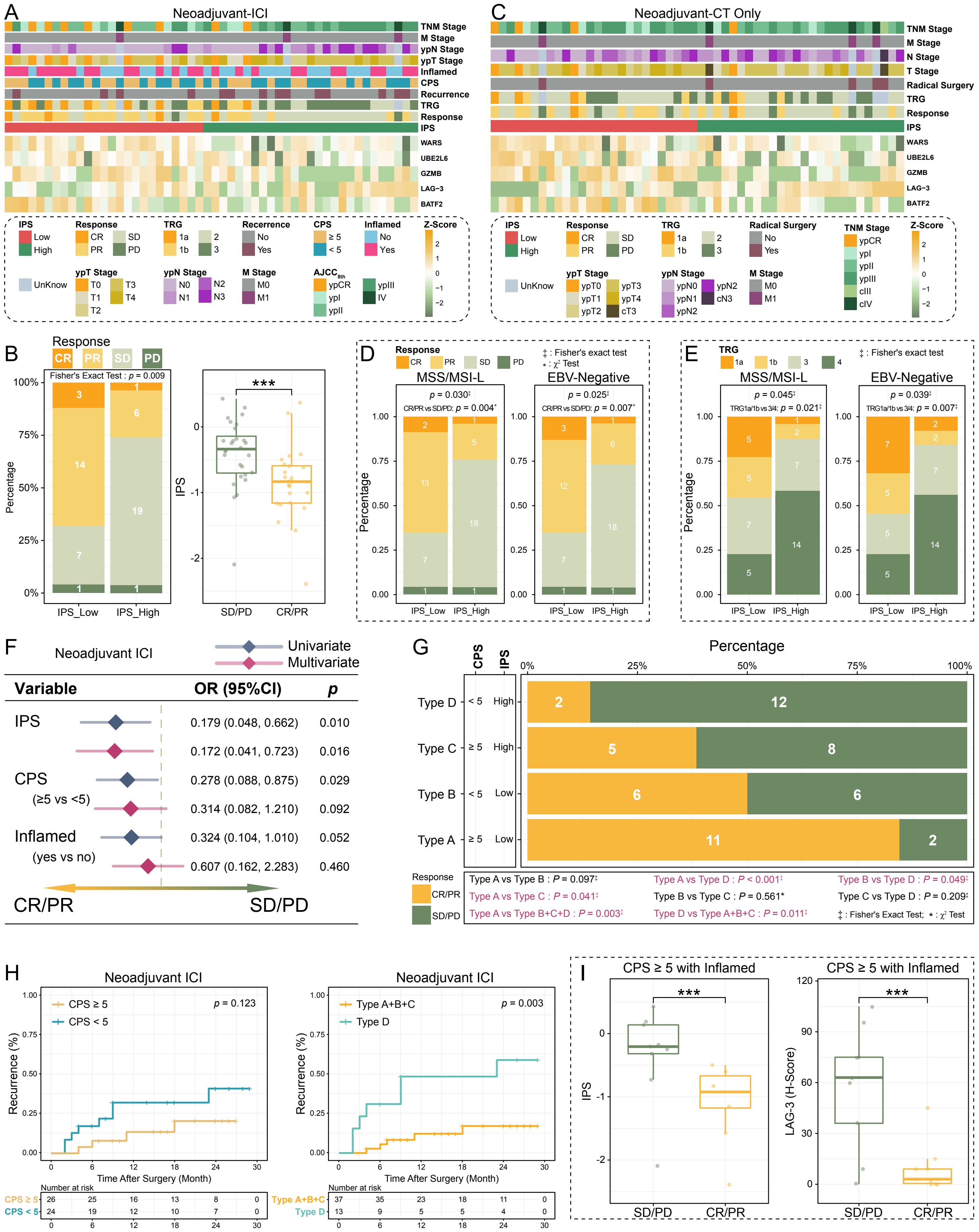

Figure S10. The IPS is a potential biomarker for predicting ICI therapy. (**A**) Evaluation of the IPS in patients receiving neoadjuvant ICI therapy (n = 52). (**B**) Composition of response to neoadjuvant ICI therapy in IPS^{Low} (n = 24) versus IPS^{High} (n = 26; *p* = 0.009, Fisher's exact test). Meanwhile, the IPS was compared between responding and non-responding patients (*p* < 0.001, Mann–Whitney U-test). (**C**) Evaluation of the IPS in patients receiving neoadjuvant chemotherapy only (n = 52). (**D**) Composition of the response to neoadjuvant ICI therapy in patients with GC with IPS^{Low} vs IPS^{High} in MSS/MSI-L (n = 48) or EBV-negative (n = 49) subtypes. (**E**) Composition of tumor regression grade (TRG) to neoadjuvant ICI therapy in patients with GC with IPS^{Low} vs IPS^{High} in MSS/MSI-L (n = 46) or EBV-negative subtypes (n = 47). (**F**) Univariate and multivariate logistic regression analysis to confirm the value of biomarkers (IPS, CPS, and Inflamed phenotype) for predicting neoadjuvant ICI therapy (outcome: CR/PR vs SD/PD). OR: Odd Ratio. (**G**) Comparison of the objective response rate (ORR) to neoadjuvant ICI therapy across Type A (IPS^{Low} with CPS ≥ 5), Type B (IPS^{Low} with CPS < 5), Type C (IPS^{High} with CPS ≥ 5), and Type D (IPS^{High} with CPS < 5). (**H**) Kaplan–Meier survival analysis demonstrates that IPS combined with CPS better predicted postoperative recurrence in patients receiving ICI therapy than CPS only (log-rank test). (**I**) Comparison of IPS and LAG-3 in responsive (CR/PR, n = 10) and non-responsive (SD/PD, n = 9) patients with GC with CPS ≥ 5 and the Inflamed phenotype (n^{Total} = 19). ***p < 0.001, Mann–Whitney U-test. The thick line shows the median value. The bottom and top of the boxes are the 25th and 75th percentile (interquartile range) and extend through the whiskers to 1.5 times the interquartile range.

Figure S11

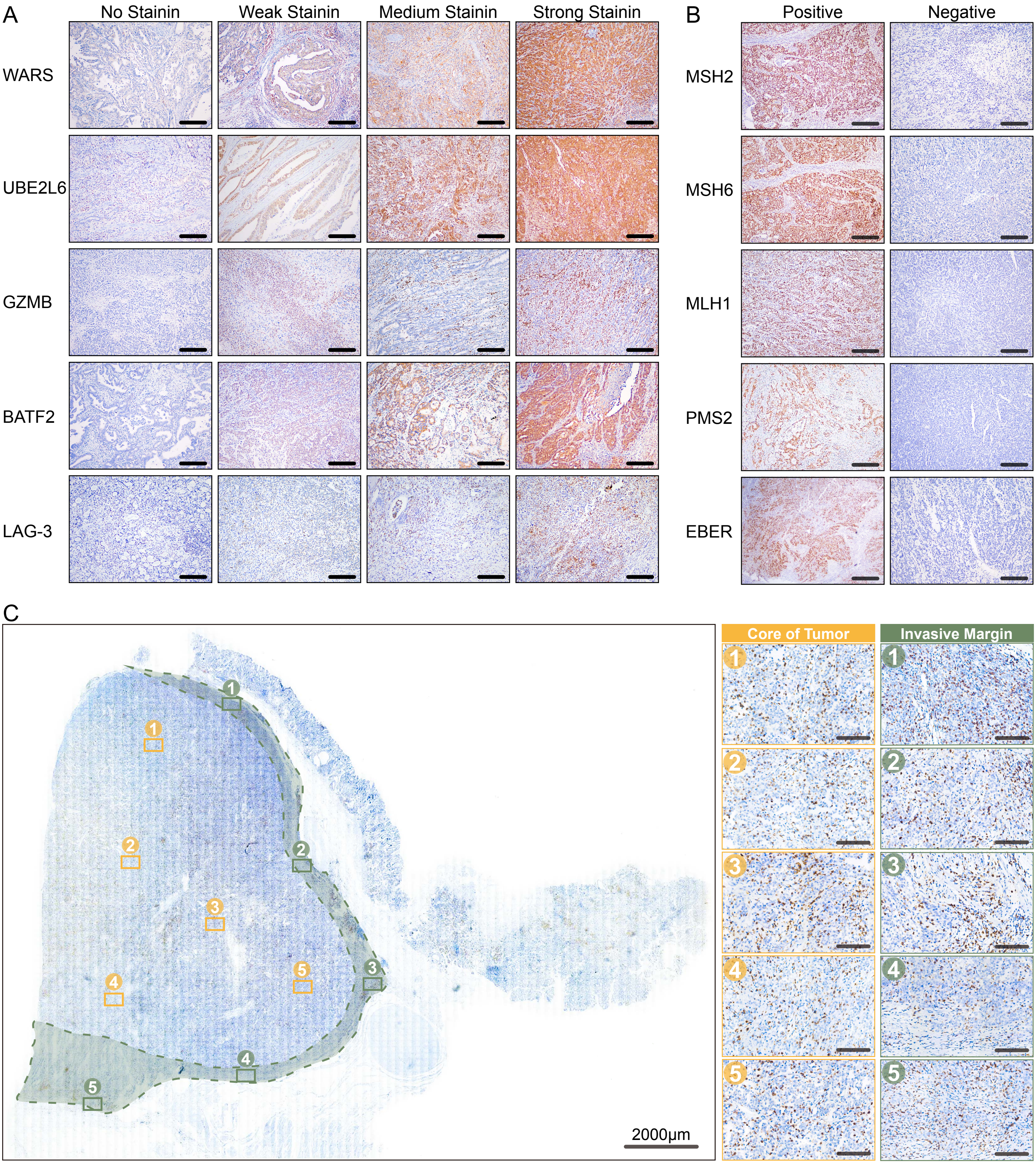

Figure S11. Evaluation criteria for immunohistochemistry and in situ hybridization. (**A**) WARS, UBE2L6, GZMB, BATF2, and LAG-3. (**B**) microsatellite instability (MSI) status and Epstein–Barr virus (EBV) status. Scale bar = 200 μm. (**C**) Schematic diagram of quantitative analysis of immuno-markers obtained from immunohistochemical staining in GC tissues. Yellow represents the core of tumor (CT) and the green represents the invasive margin (IM). The average density of five representative CT and IM regions was considered the density of this tumor. Scale bar = 200 μm (right panel).

Table S1. Clinicopathological Characteristics of Patients with GC According to IPS in Training Cohort.

Characteristic	Training Cohort (n=506)				P [a)]
	IPS^{Low} (n=227)		IPS^{High} (n=279)		
	n	%	n	%	
**Age**					0.864 [b)]
Means ± SD	61.22±11.50		61.5±10.73		
**Sex**					0.858
Male	170	74.89%	207	74.19%	
Female	57	25.11%	72	25.81%	
**BMI**					0.471
≤ 25	184	81.06%	233	83.51%	
> 25	43	18.94%	46	16.49%	
**Resection Type**					0.250
Part gastrectomy	101	44.49%	110	39.43%	
Total gastrectomy	126	55.51%	169	60.57%	
**Tumor Size**					0.007
≤ 50mm	12	53.30%	115	41.22%	
> 50mm	106	46.70%	164	58.78%	
**Tumor Location**					0.162
Cardia	55	24.23%	53	19.00%	
Body	48	21.15%	62	22.22%	
Antrum	97	42.73%	113	40.50%	
Mix	27	11.89%	51	18.28%	
**Differentiation**					0.236
Well and middle	71	31.28%	74	26.52%	
Poor and underdifferentiated	151	66.52%	199	71.33%	
Unknow	5	2.20%	6	2.15%	
**pT Stage**					< 0.001
T1	37	16.30%	32	11.47%	
T2	30	13.22%	10	3.58%	
T3	83	36.56%	107	38.35%	
T4	77	33.92%	130	46.59%	
**pN Stage**					< 0.001
N0	68	29.96%	55	19.71%	
N1	37	16.30%	17	6.09%	
N2	53	23.35%	67	24.01%	
N3	69	30.40%	140	50.18%	
**pTNM Stage**					< 0.001
I	51	22.47%	31	11.11%	
II	51	22.47%	42	15.05%	
III	125	55.07%	206	73.84%	
**MSI Status**					0.047
MSI-H (dMMR)	26	11.45%	18	6.45%	
MSS/MSI-L (pMMR)	201	88.55%	261	93.55%	
**EBV Status**					< 0.001
Positive	32	14.10%	10	3.58%	
Negative	195	85.90%	269	96.42%	
**Adjuvant Chemotherapy**					0.396
Yes	154	67.84%	199	71.33%	
No	73	32.16%	80	28.67%	
**CEA**					0.055
Normal	191	84.14%	216	77.42%	
Elevated	36	15.86%	63	22.58%	
**CA19-9**					0.248
Normal	192	84.58%	221	79.21%	
Elevated	35	15.42%	58	20.79%	

[a)] calculated by Chi-Square test; [b)] calculated by Mann-Whitney U test.

Table S2. Clinicopathological Characteristics of Patients with GC According to IPS in the External Cohorts.

Characteristic	Central China Cohort					North China Cohort					South China Cohort				
	IPS^{Low}		IPS^{High}		P [a)]	IPS^{Low}		IPS^{High}		P [a)]	IPS^{Low}		IPS^{High}		P [a)]
	n	%	n	%		n	%	n	%		n	%	n	%	
**Total Patients**	130		148			94		102			81		85		
**Age (Mean±SD)**	62.02±9.89		61.66±11.19		0.773 [b)]	64.04±9.78		63.24±1.50		0.449 [b)]	58.77±12.04		61.62±12.13		0.232 [b)]
**Sex**					0.611					0.141					0.394
Male	94	72.31%	111	75.00%		58	61.70%	73	71.57%		51	62.96%	48	56.47%	
Female	36	27.69%	37	25.00%		35	37.23%	28	27.45%		30	37.04%	37	43.53%	
**BMI**					0.089					0.136					0.298
≤ 25	14	10.77%	7	4.73%		65	69.15%	80	78.43%		60	74.07%	71	83.53%	
> 25	34	26.15%	42	28.38%		28	29.79%	21	20.59%		15	18.52%	9	10.59%	
**Resection Type**					0.914					0.316					0.290
Part gastrectomy	86	66.15%	97	65.54%		67	71.28%	66	64.71%		60	74.07%	68	80.00%	
Total gastrectomy	44	33.85%	51	34.46%		26	27.66%	35	34.31%		21	25.93%	16	18.82%	
**Tumor Size**					0.142					0.575					0.239
≤ 50mm	90	69.23%	114	77.03%		60	63.83%	69	67.65%		60	74.07%	55	64.71%	
> 50mm	40	30.77%	34	22.97%		33	35.11%	32	31.37%		17	20.99%	24	28.24%	
**Tumor Location**					0.253					0.543					0.254
Cardia	41	31.54%	53	35.81%		16	17.02%	22	21.57%		12	14.81%	5	5.88%	
Body	29	22.31%	34	22.97%		8	8.51%	8	7.84%		18	22.22%	16	18.82%	
Antrum	36	27.69%	46	31.08%		59	62.77%	55	53.92%		50	61.73%	61	71.76%	
Mix	24	18.46%	15	10.14%		10	10.64%	16	15.69%		1	1.23%	1	1.18%	
**Differentiation**					0.693					-					0.059
Well and middle	81	62.31%	89	60.14%		-	-	-	-		37	45.68%	28	32.94%	
Poor and underdifferentiated	47	36.15%	57	38.51%		-	-	-	-		41	50.62%	57	67.06%	
**pT Stage**					0.170					0.052					0.210
T1	17	13.08%	10	6.76%		10	10.64%	7	6.86%		13	16.05%	6	7.06%	
T2	22	16.92%	20	13.51%		13	13.83%	4	3.92%		17	20.99%	14	16.47%	
T3	12	9.23%	11	7.43%		23	24.47%	33	32.35%		16	19.75%	19	22.35%	
T4	79	60.77%	107	72.30%		47	50.00%	57	55.88%		35	43.21%	46	54.12%	
**pN Stage**					0.012					0.134					0.038

N0	61	46.92%	50	33.78%		34	36.17%	24	23.53%		36	44.44%	23	27.06%	
N1	31	23.85%	29	19.59%		19	20.21%	17	16.67%		14	17.28%	18	21.18%	
N2	28	21.54%	41	27.70%		15	15.96%	24	23.53%		16	19.75%	14	16.47%	
N3	10	7.69%	28	18.91%		22	23.40%	36	35.29%		15	18.52%	30	35.29%	
**pTNM Stage**					0.046					0.017					0.024
I	35	26.92%	22	14.86%		20	21.28%	8	7.84%		23	28.40%	11	12.94%	
II	23	17.70%	30	20.27%		22	23.40%	22	21.57%		23	28.40%	22	25.88%	
III	72	55.38%	96	64.86%		57	60.64%	71	69.61%		35	43.21%	52	61.18%	
**Adjuvant Chemotherapy**					0.300					0.092					0.982
Yes	62	47.69%	63	42.57%		44	46.81%	60	58.82%		56	69.14%	59	69.41%	
No	24	18.46%	34	22.97%		49	52.13%	41	40.20%		22	27.16%	23	27.06%	
**CEA**					0.263					0.181					0.692‡
Normal	93	71.54%	103	69.59%		29	30.85%	24	23.53%		28	34.57%	38	44.71%	
Elevated	21	16.15%	33	22.30%		4	4.26%	8	7.84%		2	2.47%	5	5.88%	
**CA19-9**					0.072					0.958					0.223 [c)]
Normal	53	40.77%	47	31.76%		28	29.79%	27	26.47%		21	25.93%	27	31.76%	
Elevated	5	3.85%	12	8.11%		5	5.32%	5	4.90%		1	1.23%	6	7.06%	

[a)] calculated by Chi-Square test; [b)] calculated by Mann-Whitney U test; [c)] calculated by Fisher's exact test.

Table S3. Comparing the Efficacy of Neoadjuvant Chemotherapy in the patients with GC with IPS^{Low} versus IPS^{High}.

**Characteristic**	**IPS^{Low}**		**IPS^{High}**		***P***
	**n**	**%**	**n**	**%**	
**Total Patients**	26		26		
**Response**					0.244 [a)]
CR/PR	11	42.31%	7	26.92%	
SD/PD	15	57.69%	19	73.08%	
**TRG**					0.727 [b)]
1a/1b	6	23.08%	4	15.38%	
2/3	19	73.08%	19	73.08%	
**ypT Stage**					0.725 [b)]
T0/T1	4	15.38%	5	19.23%	
T2/T3	21	80.77%	19	73.08%	
**ypN Stage**					0.176 [a)]
N0	12	46.15%	7	26.92%	
N1-N3	13	50.00%	17	65.38%	
**pTNM Stage**					0.189 [b)]
pCR/I	7	26.92%	3	11.54%	
II/III	18	69.23%	21	80.77%	

[a)] calculated by Chi-Square test; [b)] calculated by Fisher's exact test.

Table S4. Comparing the Efficacy of Neoadjuvant ICI Therapy Combined with Chemotherapy versus Neoadjuvant Chemotherapy Only in the Patients with GC According to IPS.

Characteristic	IPS^Low					IPS^High				
	Neoadjuvant ICI with CT		Neoadjuvant CT Only		*P* [a]	Neoadjuvant ICI with CT		Neoadjuvant CT Only		*P* [a]
	n	%	n	%		n	%	n	%	
**Total Patients**	25		26			27		26		
**Response**					0.065					0.934
CR/PR	17	68.00%	11	42.31%		7	25.93%	7	26.92%	
SD/PD	8	32.00%	15	57.69%		20	74.07%	19	73.08%	
**TRG**					0.059					0.850 [b]
1a/1b	12	48.00%	6	23.08%		4	14.81%	4	15.38%	
2/3	12	48.00%	19	73.08%		22	81.48%	19	73.08%	
**ypT Stage**					0.032 [b]					0.725 [b]
T0/T1	11	44.00%	4	15.38%		4	14.81%	5	19.23%	
T2/T3	13	52.00%	21	80.77%		21	77.78%	19	73.08%	
**ypN Stage**					0.052					0.830
N0	18	72.00%	12	46.15%		8	29.63%	7	26.92%	
N1-N3	6	24.00%	13	50.00%		17	62.96%	17	65.38%	
**pTNM**					0.083					1.000 [b]
pCR/I	13	52.00%	7	26.92%		4	14.81%	3	11.54%	
II/III	12	48.00%	18	69.23%		23	85.19%	21	80.77%	

[a] calculated by Chi-Square test; [b] calculated by Fisher's exact test.

Table S5. Clinicopathological Characteristics of Patients with GC in Training Cohorts (FMUUN).

Characteristic	Training Cohort (n=506)				P [a)]
	Discovery Cohort (n=253)		Remaining Patients (n=253)		
	n	%	n	%	
**Age**					0.638 [b)]
Mean ± SD	60.8±11.4		61.4±10.9		
**Sex**					0.760
Male	190	75.10%	187	73.91%	
Female	63	24.90%	66	26.09%	
**BMI**					0.726
≤ 25	210	83.00%	207	81.82%	
> 25	43	17.00%	46	18.18%	
**Resection Type**					0.207
Part gastrectomy	113	44.66%	98	38.73%	
Total gastrectomy	140	55.34%	155	61.27%	
**Tumor Size**					0.593
≤ 50mm	121	47.83%	115	45.45%	
> 50mm	132	52.17%	138	54.55%	
**Tumor Location**					0.081
Cardia	58	22.92%	50	19.76%	
Body	44	17.39%	66	26.08%	
Antrum	114	45.06%	96	37.94%	
Mix	37	14.62%	41	16.21%	
**Differentiation**					0.844
Well and middle	72	28.46%	73	28.85%	
Poor and undifferentiated	178	70.36%	172	67.98%	
Unknow	3	1.18%	8	3.17%	
**pT Stage**					0.832
T1	31	12.25%	38	15.02%	
T2	21	8.30%	19	7.51%	
T3	96	37.94%	94	37.15%	
T4	105	41.50%	102	40.32%	
**pN Stage**					0.311
N0	60	23.72%	63	24.90%	
N1	33	13.04%	21	8.30%	
N2	65	25.69%	65	25.69%	
N3	105	41.50%	104	41.11%	
**pTNM Stage**					0.512
I	38	15.02%	44	17.39%	
II	51	20.16%	42	16.60%	
III	164	64.82%	167	66.01%	
**MSI Status**					0.528
MSI-H (dMMR)	20	7.91%	24	9.49%	
MSS/MSI-L (pMMR)	233	92.09%	229	90.51%	
**EBV Status**					1.000
Positive	21	8.30%	21	8.30%	
Negative	232	91.70%	232	91.70%	
**Adjuvant Chemotherapy**					0.498
Yes	180	71.15%	173	68.38%	
No	73	28.85%	80	31.62%	
**CEA**					0.911
Normal	203	80.24%	204	80.63%	
Elevated	50	19.76%	49	19.37%	
**CA19-9**					0.422
Normal	203	80.24%	210	83.00%	
Elevated	50	19.76%	43	17.00%	

[a)] calculated by Chi-Square test; [b)] calculated by Mann-Whitney U test.

Table S6. Clinicopathological Characteristics of Patients with GC in the External Cohorts.

Characteristic	Central China Cohort						North China Cohort						South China Cohort					
	USTCFAH		BMCFAH		Total		JUBFAH		LCH		Total		KMUFAH		GMUATH		total	
	n	%	n	%	n	%	n	%	n	%	n	%	n	%	n	%	n	%
**Total Patients**	181		97		278		96		98		194		106		60		166	
**Age (Mean±SD)**	60.69±10.92		63.96±9.64		61.83±10.59		63.94±11.32		63.32±8.89		61.60±11.55%		63.07±11.27		55.22±12.07		60.63±12.13	
**Sex**																		
Male	131	72.38%	74	76.29%	205	73.74%	61	63.54%	70	71.43%	131	67.53%	63	59.43%	36	60.00%	99	59.64%
Female	50	27.62%	23	23.71%	73	26.26%	35	36.46%	28	28.57%	63	32.47%	43	40.57%	24	40.00%	67	40.36%
**BMI**																		
≤ 25	-	-	76	78.35%	76	27.34%	82	85.42%	63	64.29%	145	74.74%	84	79.25%	47	78.33%	131	78.92%
> 25	-	-	21	21.65%	21	7.55%	14	14.58%	35	35.71%	49	25.26%	11	10.38%	13	21.67%	24	14.46%
**Resection Type**																		
Part gastrectomy	103	56.91%	80	82.47%	183	65.83%	71	73.96%	62	63.27%	133	68.56%	77	72.64%	51	85.00%	128	77.11%
Total gastrectomy	78	43.09%	17	17.53%	95	34.17%	25	26.04%	36	36.73%	61	31.44%	28	26.42%	9	15.00%	37	22.29%
**Tumor Size**																		
≤ 50mm	127	70.17%	77	79.38%	204	73.38%	55	57.29%	71	72.45%	126	64.95%	28	26.42%	13	21.67%	41	24.70%
> 50mm	54	29.83%	20	20.62%	74	26.62%	41	42.71%	24	24.49%	65	33.51%	70	66.04%	45	75.00%	115	69.28%
**Tumor Location**																		
Cardia	56	30.94%	38	39.18%	94	33.81%	19	19.79%	19	19.39%	38	19.59%	12	11.32%	5	8.33%	17	10.24%
Body	54	29.83%	9	9.28%	63	22.66%	13	13.54%	3	3.06%	16	8.25%	19	17.92%	15	25.00%	34	20.48%
Antrum	68	37.57%	14	14.43%	82	29.50%	64	66.67%	50	51.02%	114	58.76%	72	67.92%	39	65.00%	111	66.87%
Mix	3	1.66%	36	37.11%	39	14.03%	0	0.00%	26	26.53%	26	13.40%	1	0.94%	1	1.67%	2	1.20%
**Differentiation**																		
Well and middle	96	53.04%	74	76.29%	170	61.15%	-	-	-	-	-	-	54	50.94%	11	18.33%	65	39.16%
Poor and underdifferentiated	81	44.75%	23	23.71%	104	37.41%	-	-	-	-	-	-	51	48.11%	47	78.33%	98	59.04%
**pT Stage**																		
T1	20	11.05%	7	7.22%	27	9.71%	5	5.21%	12	12.24%	17	8.76%	13	12.26%	6	10.00%	19	11.45%
T2	31	17.13%	11	11.34%	52	18.71%	4	4.17%	13	13.27%	17	8.76%	19	17.92%	12	20.00%	31	18.67%
T3	0	0.00%	23	23.71%	23	8.27%	46	47.92%	10	10.20%	56	28.87%	29	27.36%	6	10.00%	35	21.08%

T4	130	71.82%	56	57.73%	186	66.91%	41	42.71%	63	64.29%	104	53.61%	45	42.45%	36	60.00%	81	48.80%
**pN Stage**																		
N0	72	39.78%	39	40.21%	111	39.93%	15	15.63%	43	43.88%	58	29.90%	36	33.96%	23	38.33%	59	35.54%
N1	35	19.34%	25	25.77%	60	21.58%	17	17.71%	19	19.39%	36	18.56%	21	19.81%	11	18.33%	32	19.28%
N2	41	22.65%	28	28.87%	69	24.82%	18	18.75%	21	21.43%	39	20.10%	17	16.04%	13	21.67%	30	18.07%
N3	33	18.23%	5	5.15%	38	13.67%	46	47.92%	15	15.31%	61	31.44%	32	30.19%	13	21.67%	45	27.11%
**pTNM Stage**																		
I	45	24.86%	12	12.37%	57	20.50%	8	8.33%	20	20.41%	28	14.43%	23	21.70%	11	18.33%	34	20.48%
II	28	15.50%	25	25.77%	53	19.07%	19	19.79%	25	25.51%	44	22.68%	30	28.30%	15	25.00%	45	27.11%
III	108	59.67%	60	61.86%	168	60.43%	69	71.88%	53	54.08%	122	62.89%	53	50.00%	34	56.67%	87	52.41%
**Adjuvant Chemotherapy**																		
Yes	52	28.73%	73	75.26%	125	44.96%	31	32.29%	73	74.49%	104	53.61%	65	61.32%	50	83.33%	115	69.28%
No	37	20.44%	21	21.65%	58	20.86%	65	67.71%	25	25.51%	90	46.39%	35	33.02%	10	16.67%	45	27.11%
**CEA**																		
Normal	114	62.98%	82	84.54%	196	70.50%	-	-	53	54.08%	53	27.32%	66	62.26%	-	-	66	39.76%
Elevated	39	21.55%	15	15.46%	54	19.42%	-	-	12	12.24%	12	6.19%	7	6.60%	-	-	7	4.22%
**CA19-9**																		
Normal	16	8.84%	84	86.60%	100	35.97%	-	-	10	10.20%	10	5.15%	48	45.28%	-	-	48	28.92%
Elevated	4	2.21%	13	13.40%	17	6.12%	-	-	55	56.12%	55	28.35%	7	6.60%	-	-	7	4.22%

Table S7. Clinicopathological Characteristics of Patients with GC in the FMUUN-RNA_Seq Cohort (n = 79).

ID	Sex	Age	Histopathological diagnosis	pT	pN	pM	pTNM	Location	Sequencing
991161	Male	65	Adenocarcinoma	T3	N2	M0	III	Cardia	WTS
829840	Male	51	Adenocarcinoma	T4	N0	M0	II	Body	WTS
993850	Male	54	Adenocarcinoma	T4	N2	M0	III	Antrum	WTS
994572	Male	71	Adenocarcinoma	T4	N2	M0	III	Antrum	WTS
994420	Male	79	Adenocarcinoma	T3	N2	M0	III	Cardia	WTS
994581	Female	55	Adenocarcinoma	T3	N3	M0	III	Body	WTS
995217	Male	65	Adenocarcinoma	T3	N2	M0	III	Cardia	WTS
995179	Female	73	Adenocarcinoma	T2	N2	M0	II	Body	WTS
995326	Female	69	Adenocarcinoma	T2	N0	M0	I	Antrum	WTS
995637	Male	67	Adenocarcinoma	T4	N3	M0	III	Body	WTS
1002825	Male	52	Adenocarcinoma	T3	N0	M0	II	Cardia	WTS
1003991	Male	74	Adenocarcinoma	T1	N0	M0	I	Cardia	WTS
1005136	Male	57	Adenocarcinoma	T3	N3	M0	III	Antrum	WTS
772745	Male	68	Adenocarcinoma	T3	N2	M0	III	Antrum	WTS
1006613	Male	65	Adenocarcinoma	T4	N3	M0	III	Antrum	WTS
1007099	Male	72	Adenocarcinoma	T3	N3	M0	III	Body	WTS
1006443	Male	61	Adenocarcinoma	T3	N3	M0	III	Cardia	WTS
1005701	Male	44	Adenocarcinoma	T3	N2	M0	III	Antrum	WTS
1010626	Female	72	Adenocarcinoma	T4	N3	M0	III	Body	WTS
1010839	Female	60	Adenocarcinoma	T4	N2	M0	III	Mix	WTS
1011934	Male	66	Adenocarcinoma	T4	N3	M0	III	Cardia	WTS
835615	Male	66	Adenocarcinoma	T3	N2	M0	III	Cardia	WTS
1012937	Female	58	Adenocarcinoma	T3	N0	M0	II	Cardia	WTS
1015114	Male	74	Adenocarcinoma	T3	N1	M0	II	Cardia	WTS
1017741	Male	71	Adenocarcinoma	T2	N1	M0	II	Cardia	WTS
1017645	Male	73	Adenocarcinoma	T3	N0	M0	II	Antrum	WTS
1018346	Male	64	Adenocarcinoma	T3	N2	M0	III	Antrum	WTS
1018734	Male	64	Adenocarcinoma	T3	N3	M0	III	Cardia	WTS
1018645	Male	82	Adenocarcinoma	T3	N0	M0	II	Cardia	WTS
1019611	Male	54	Adenocarcinoma	T3	N3	M0	III	Cardia	WTS
1024826	Male	74	Adenocarcinoma	T3	N3	M0	III	Cardia	WTS
1007072	Female	63	Adenocarcinoma	T4	N1	M0	III	Body	WTS
1138443	Male	65	Adenocarcinoma	T3	N2	M0	III	Antrum	WTS + WES
1134739	Female	36	Adenocarcinoma	T3	N3	M0	III	Antrum	WTS + WES
1138612	Female	62	Adenocarcinoma	T3	N3	M0	III	Antrum	WTS + WES
1145704	Male	71	Adenocarcinoma	T3	N2	M0	III	Antrum	WTS + WES
1147375	Male	62	Adenocarcinoma	T4	N2	M0	III	Cardia	WTS + WES
1139153	Male	64	Adenocarcinoma	T3	N0	M0	II	Antrum	WTS + WES
1139908	Female	36	Adenocarcinoma	T4	N3	M0	III	Cardia	WTS + WES
1147432	Male	53	Adenocarcinoma	T1	N0	M0	I	Antrum	WTS + WES
1140888	Male	65	Adenocarcinoma	T3	N3	M0	III	Cardia	WTS + WES
1136578	Male	66	Adenocarcinoma	T4	N1	M0	III	Mix	WTS + WES
1147802	Female	72	Adenocarcinoma	T3	N3	M0	III	Antrum	WTS + WES
1148300	Female	63	Adenocarcinoma	T4	N3	M0	III	Body	WTS + WES
1140020	Male	68	Adenocarcinoma	T4	N1	M0	III	Cardia	WTS + WES
1140705	Male	66	Adenocarcinoma	T2	N0	M0	I	Cardia	WTS + WES
1138324	Male	55	Adenocarcinoma	T3	N1	M0	II	Antrum	WTS + WES
1140597	Female	37	Adenocarcinoma	T4	N3	M0	III	Cardia	WTS + WES
1133958	Male	76	Adenocarcinoma	T3	N0	M0	II	Mix	WTS + WES
1138167	Male	71	Adenocarcinoma	T1	N0	M0	I	Antrum	WTS + WES
1132994	Male	49	Adenocarcinoma	T3	N2	M0	III	Antrum	WTS + WES
1146331	Female	57	Adenocarcinoma	T4	N3	M0	III	Antrum	WTS + WES
1146501	Male	55	Adenocarcinoma	T4	N0	M0	II	Body	WTS + WES
1137201	Male	37	Adenocarcinoma	T4	N3	M0	III	Cardia	WTS + WES
1146085	Male	44	Adenocarcinoma	T4	N3	M0	III	Antrum	WTS + WES
1139708	Male	77	Adenocarcinoma	T3	N0	M0	II	Cardia	WTS + WES
1139578	Male	70	Adenocarcinoma	T3	N0	M0	II	Cardia	WTS + WES
1145167	Male	52	Adenocarcinoma	T4	N2	M0	III	Antrum	WTS + WES
1140957	Male	56	Adenocarcinoma	T4	N3	M0	III	Cardia	WTS + WES
1147455	Male	60	Adenocarcinoma	T2	N0	M0	I	Antrum	WTS + WES
1145920	Male	57	Adenocarcinoma	T4	N1	M0	III	Cardia	WTS + WES
1136922	Male	55	Adenocarcinoma	T4	N3	M0	III	Mix	WTS + WES
1147277	Male	55	Adenocarcinoma	T3	N2	M0	III	Cardia	WTS + WES
1134718	Female	72	Adenocarcinoma	T1	N1	M0	I	Antrum	WTS + WES
1146048	Male	74	Adenocarcinoma	T3	N2	M0	III	Mix	WTS + WES
1137766	Female	26	Adenocarcinoma	T4	N2	M0	III	Cardia	WTS + WES
1134346	Male	55	Adenocarcinoma	T2	N0	M0	I	Body	WTS + WES
1134368	Male	65	Adenocarcinoma	T3	N3	M0	III	Antrum	WTS + WES
1141994	Male	66	Adenocarcinoma	T3	N3	M0	III	Antrum	WTS + WES
1134882	Male	65	Adenocarcinoma	T3	N2	M0	III	Cardia	WTS + WES
1145564	Female	55	Adenocarcinoma	T4	N3	M0	III	Cardia	WTS + WES
1134924	Male	62	Adenocarcinoma	T3	N1	M0	II	Cardia	WTS + WES
1136520	Female	54	Adenocarcinoma	T4	N1	M0	III	Cardia	WTS + WES
1135146	Female	64	Adenocarcinoma	T1	N1	M0	I	Antrum	WTS + WES
1144293	Male	61	Adenocarcinoma	T3	N2	M0	III	Cardia	WTS + WES
1136058	Female	45	Adenocarcinoma	T4	N2	M0	III	Antrum	WTS + WES
1146129	Male	56	Adenocarcinoma	T1	N2	M0	II	Antrum	WTS + WES
1134902	Male	72	Adenocarcinoma	T4	N3	M0	III	Body	WTS + WES
1145207	Male	57	Adenocarcinoma	T4	N3	M0	III	Antrum	WTS + WES

WTS: Whole-Transcriptome sequencing; **WES**: Whole-exome sequencing.

Table S8. Clinicopathological Characteristics of the Patients with GC Receiving Neoadjuvant Therapy.

Characteristic	Neoadjuvant ICI Therapy with Chemotherapy		Neoadjuvant Chemotherapy only		*P*
	n	%	n	%	
**Total Patients**	52		52		
**Age**					0.282 [b)]
Mean ± SD	61.23 ± 10.19		59.24 ± 10.30		
**Sex**					0.819 [a)]
Male	40	76.92%	39	75.00%	
Female	12	23.08%	13	25.00%	
**Neoadjuvant Cycle**					0.326 [a)]
≤ 3	27	51.92%	22	42.31%	
> 3	25	48.08%	30	57.69%	
**Tumor Size**					0.486 [a)]
≤ 5cm	26	50.00%	30	57.69%	
> 5cm	23	44.23%	20	38.46%	
**Tumor Location**					0.174 [a)]
Cardia	17	32.69%	27	51.92%	
Body	12	23.08%	8	15.38%	
Antrum	6	11.54%	7	13.46%	
Mix	17	32.69%	10	19.23%	
**Differentiation**					0.507 [a)]
Well and middle	20	38.46%	23	44.23%	
Poor and underdifferentiated	21	40.38%	18	34.62%	
**Resection Type**					0.317 [c)]
Part gastrectomy	7	13.46%	3	5.77%	
Total gastrectomy	42	80.77%	46	88.46%	
**Radical Resection**					0.715 [c)]
Yes	49	94.23%	47	90.38%	
No	3	5.77%	5	9.62%	
**TRG**					0.263 [c)]
TRG 1a	9	17.31%	4	7.69%	
TRG 1b	7	13.46%	6	11.54%	
TRG 2	15	28.85%	23	44.23%	
TRG 3	19	36.54%	15	28.85%	
**ypT Stage**					0.622 [c)]
T0	8	15.38%	4	7.69%	
T1	6	11.54%	5	9.62%	
T2	5	9.62%	4	7.69%	
T3	19	36.54%	26	50.00%	
T4	10	19.23%	10	19.23%	
**ypN Stage**					0.229 [c)]
N0	25	48.08%	19	36.54%	
N1	19	36.54%	12	23.08%	
N2	3	5.77%	9	17.31%	
N3	11	21.15%	9	17.31%	
**TNM Stage**					0.536 [c)]
ypCR	9	17.31%	4	7.69%	
ypI	8	15.38%	6	11.54%	
ypII	16	30.77%	17	32.69%	
ypIII	16	30.77%	21	40.38%	
cIV	3	5.77%	4	7.69%	
**Radiological Response**					0.364 [c)]
CR	4	7.69%	2	3.85%	
PR	20	38.46%	16	30.77%	
SD	26	50.00%	28	53.85%	
PD	2	3.85%	6	11.54%	
**MSI Status**					-
MSI-H (dMMR)	4	7.69%	-	-	
MSS/MSI-L (pMMR)	48	92.31%	-	-	
**EBV Status**					-
Positive	3	5.77%	-	-	
Negative	49	94.23%	-	-	

[a)] calculated by Chi-Square test; [b)] calculated by Mann-Whitney U test; [c)] calculated by Fisher's exact test.

Table S9. Antibody Sources for Immunohistochemical and Multiplex immunohistochemistry staining.

Markers	Dilution	Antibody Source		Application	Markers	Dilution	Antibody Source		Application
IFNG	1:200	Abcam	ab218426	IHC	NKG7	1:200	GeneTex	GTX01839	IHC
GZMB	1:400	Abcam	ab4059	IHC	KLRC3	1:200	Bioss	bs-16781R	IHC
WARS	1:4000	Abcam	ab109213	IHC	CD38	1:100	Affitiny	DF6551	IHC
LAG-3	1:400	Abcam	ab209236	IHC	MICB	1:400	HuaBio	ER1803-95	IHC
ETV7	1:400	Abcam	ab229832	IHC	GBP1	1:3200	Abcam	ab131255	IHC
GBP4	1:800	Abcam	ab232693	IHC	CD45	1:200	Abcam	ab10558	IHC
GBP5	1:2000	Abcam	ab244422	IHC	CD3	1:150	Abcam	ab16669	IHC
IRF1	1:200	Abcam	ab243895	IHC	CD8	1:200	Abcam	ab4055	IHC
ZNF683	1:50	SIGMA-Aldrich	HPA023865	IHC	CD4	1:400	Abcam	ab183685	IHC
KIR2DL4	1:400	Abcam	ab154386	IHC	CD45RO	1:800	Abcam	ab23	IHC
ZBED2	1:400	Invitrogen	PA5-20723	IHC	FOXP3	1:200	Abcam	ab215206	IHC
CXCL11	1:800	Abclonal	A6201	IHC	PD-L1	Ready-to-use	Ventana	SP263	IHC
CXCL9	1:400	Abcam	ab137792	IHC	MLH1	1:250	Abcam	ab92312	IHC
BATF2	1:100	Santa Curz	sc-293274	IHC	MSH2	1:250	Abcam	ab52266	IHC
FASLG	1:150	HuaBio	ER1908-69	IHC	MSH6	1:250	Abcam	ab92471	IHC
CXCR6	1:200	Affitiny	DF2328	IHC	PMS2	1:250	Abcam	ab110638	IHC
KLRD1	1:1600	Proteintech	13332-1-AP	IHC	panCK	1:100	Abcam	ab7753	mIHC
PSMB9	1:32000	Abcam	ab242061	IHC	CD68	1:200	Abcam	ab213363	mIHC
GNLY	1:100	SIGMA-Aldrich	HPA058021	IHC	CD163	1:200	Abcam	ab181422	mIHC
PSMB10	1:16000	Abcam	ab183506	IHC	CD206	1:1000	Abcam	ab64693	mIHC
UBE2L6	1:4000	Abcam	ab109086	IHC	INOS	1:200	Abcam	ab178945	mIHC
PRF1	1:50	HuaBio	ER1803-77	IHC	CD8	1:100	CST	70306S	mIHC
STAT1	1:150	Affitiny	AF6300	IHC	GZMB	1:250	Abcam	ab209236	mIHC
PSMB8	1:32000	Abcam	ab180606	IHC	EBER		ZSGB-BIO	ISH-6021	situ hybridizatio
CXCL10	1:50	R&D	MAB2662-SP	IHC					
